# One-pot synthesis of 4′-alkyl-4-cyanobiaryls on the basis of the terephthalonitrile dianion and neutral aromatic nitrile cross-coupling

**DOI:** 10.3762/bjoc.12.153

**Published:** 2016-07-25

**Authors:** Roman Yu Peshkov, Elena V Panteleeva, Wang Chunyan, Evgeny V Tretyakov, Vitalij D Shteingarts

**Affiliations:** 1 Laboratory of the Investigation of Nucleophilic and Radical Ionic Reactions, N.N. Vorozhtsov Novosibirsk Institute of Organic Chemistry of Siberian Branch of Russian Academy of Sciences, Ac. Lavrentiev Avenue, 9, Novosibirsk, 630090, Russia; 2 Natural Sciences Department, Novosibirsk State University, Pirogova St., 2, Novosibirsk, 630090, Russia; 3 Heilongjang University, Xuefu Road, 74, Harbin, 150080, China

**Keywords:** alkylcyanobiaryls, cross-coupling, cyanoarenes, reactive intermediates, reductive alkylation

## Abstract

A convenient one-pot approach to alkylcyanobiaryls is described. The method is based on biaryl cross-coupling between the sodium salt of the terephthalonitrile dianion and a neutral aromatic nitrile in liquid ammonia, and successive alkylation of the long-lived anionic intermediate with alkyl bromides. The reaction is compatible with benzonitriles that contain methyl, methoxy and phenyl groups, fluorine atoms, and a 1-cyanonaphthalene residue. The variety of ω-substituted alkyl bromides, including an extra bromine atom, a double bond, cyano and ester groups, as well as a 1,3-dioxane fragment are suitable as alkylation reagents.

## Introduction

**Scheme 1:**
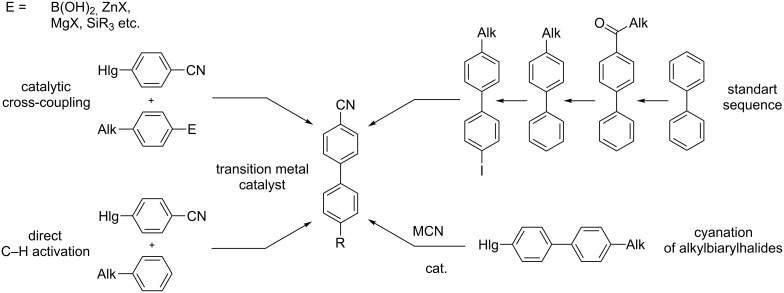
The main synthetic approaches to alkylcyanobiphenyls.

Alkylcyanobiphenyls are well known largely due to their mesogenic properties, which were discovered by Gray in the 1970^th^ [1–2]. Alkylcyanobiphenyls and their analogs (e.g., dialkyl and alkoxy alkyl derivatives) are still being used as components of liquid crystal mixtures [3–8], lubricants [9], media for conformational analysis using NMR [10–12] and EPR [13] techniques, and nanoparticles-doped liquid crystals [14]. The classic multistage approaches to these compounds, including successive biphenyl acylation–reduction–halogenation–cyanodehalogenation (Scheme 1) [15], are currently being replaced with methods based on transition-metal-catalyzed biaryl cross-coupling [16–21]. This methodology is well developed, compatible with different substituents in the substrate and opens up a new approach to a large number of functionalized biaryls with good or excellent yields. Nevertheless, the methodology has some disadvantages: the catalytic systems are quite expensive, the ligands are often hardly accessible and require special synthetic efforts, and preactivation of the substrates via introduction of an organometallic group is necessary. The last disadvantage is overcome to some extent by the direct C–H arylation protocols [18], but usually they are catalytic and thus the resulting cross-coupling products contain traces of transition metals difficult to remove that limits their use in areas such as pharmacy [22].

**Scheme 2:**
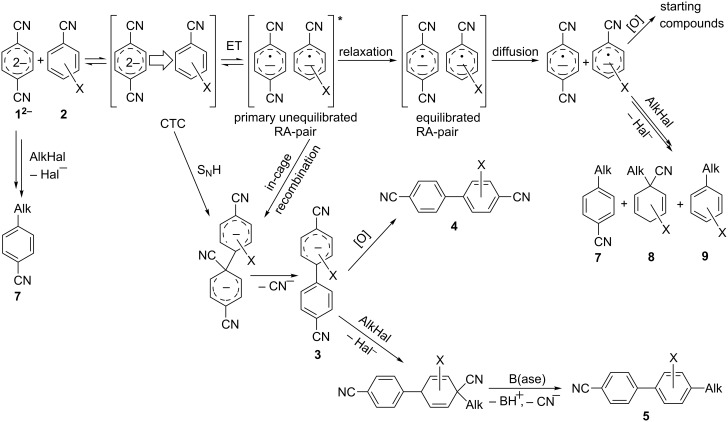
*Para*-cyanophenylation of substituted benzonitriles **2** by dianion **1^2^**^−^ with the formation of a long-lived cyanocyclohexadienyl anion **3**, followed by oxidation or alkylation, leading to dicyanobiphenyls **4** or alkylcyanobiphenyls **5**.

Because of the above-mentioned shortcomings of cross-coupling methods, we applied the method based on the direct C–H arylation of unactivated monocyanoarene **2** by the terephthalonitrile (**1^2^**^−^) dianion [23]. It was shown that the interaction of **1^2^**^−^ alkali metal salt with benzonitrile (**2a**) and its 2- and 3-CH_3_-, CH_3_O-, and F-substituted derivatives (**2b**–**f**) in liquid ammonia provided 4,4'-dicyanobiphenyls (**4**, Scheme 2) [23–24]. In the case of **1^2^**^−^ cross-coupling with 2- or 3-cyanobiphenyls, the corresponding *meta*- and *para-*dicyanoterphenyls [25] formed in good yields. The study of probable pathways for the **1^2^**^−^ interaction with benzonitriles **2** demonstrated that the cyanocyclohexadienyl anion **3** was a long-lived reaction intermediate [23–24]. This finding allowed us to obtain not only dicyanobiphenyls **4** via anion **3** oxidation, but also alkylcyanobiphenyls **5** by treating **3** with an alkyl halide. Earlier, we used butyl bromide for anion **3** trapping and obtained 4-butyl-4'-cyanobiphenyl (**5aa**) in 56% yield (Table 1, entry 1) [23]. In order to expand the scope of the synthetic utilization of the cross-coupling reaction under investigation, as well as to work out a short and convenient approach to a number of universal carbonitrilic structural blocks with different both aryl and alkyl moieties [16–19], we varied the nature of the neutral participant **2** in the coupling with dianion **1^2^**^–^ and also the reagent for anion **3** alkylation. The presented results not only support the approach based on anionic forms of cyanoarenes as effective cross-coupling reagents toward neutral unactivated substrates [26–27], but also expand the knowledge about nucleophilic mechanisms such as aromatic substitution of a hydrogen atom (S_N_H) [28] and the vicarious substitution [29].

## Results and Discussion

The suggested one-pot synthesis of alkylcyanobiaryls **5** comprises consecutive generation of terephthalonitrile dianion (**1^2–^**) [30–32] by the addition of metallic sodium to a suspension of dinitrile **1** in liquid ammonia, treating of the thus formed **1^2^**^−^ salt with a twofold excess of nitrile **2**, stirring the reaction mixture for ca. 1.5 h, which is necessary for cross-coupling, and final quenching by the addition of an excess of alkyl halide **6**. The reaction proceeds under evaporating ammonia at −33 °C, and it does not need an additional source of inert atmosphere for the overall reaction time, which is ca. 3 h. The reaction mixtures thus obtained, along with target alkylcyanobiaryls **5** (average yields 40–70%, Table 1), contain starting materials **1** (up to 10 mol %), **2** (up to 46% relative to the initial amount of **2**), and several byproducts, such as 4-alkylbenzonitriles **7**, 1-alkylcyclohexa-2,5-dienecarbonitriles **8**, and alkylbenzenes **9** (together, up to 25%).

**Table 1:** Interaction of dianion **1^2^**^−^ with benzonitrile (**2a**), followed by alkylation of **3** with ω-substituted-alkyl bromides **6a**–**f**.

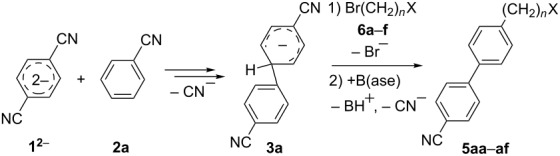				

Entry	Benzo nitrile, **2**	Alkyl halide, **6**	Alkylcyanobiphenyl product, **5**	Product yield^a^

1	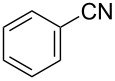 **2a**	BuBr **6a**	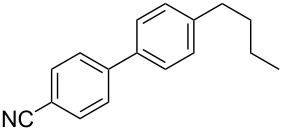 **5aa**	56^b^
2	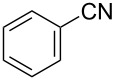 **2a**	Br(CH_2_)_3_CH=CH_2_ **6b**	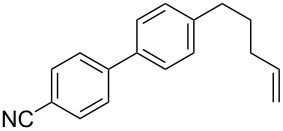 **5ab**	70 (64)
3	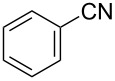 **2a**	Br(CH_2_)_5_Br **6c**	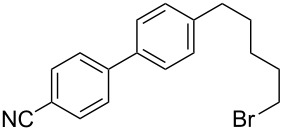 **5ac**	61 (55)
4	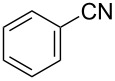 **2a**	Br(CH_2_)_4_CN **6d**	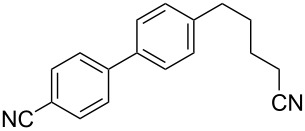 **5ad**	64 (52)
5	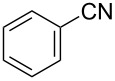 **2a**	Br(CH_2_)_5_CO_2_Et **6e**	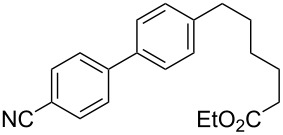 **5ae**	59 (51)
6	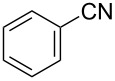 **2a**	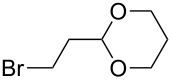 **6f**	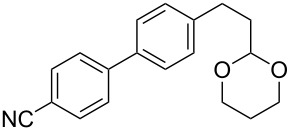 **5af**	44 (38)

^a^NMR yield, % (isolated yield, %) calculated as mean values of no less than three runs. Deviation does not exceed 5%; ^b^see [23].

According to the total reaction scheme, these byproducts arise via alkylation of the anionic forms of nitriles **1** and **2** [26]. The latter appear in the reaction media due to the electron transfer from **1^2^**^−^ to neutral cyanoarene **2** (Scheme 2). Of note, 4,4'-dicyanobiphenyl **4** (formed due to anion **3** oxidation) in all cases except one (see below) is detected in the reaction mixtures only in trace amounts (up to 5%). Thus, the alkylation step can be assumed to be quite fast, and the target product yield mostly depends on the efficiency of the cross-coupling stage. Relatively volatile components **2** and **7**–**9** are easily distilled off in vacuo, and alkylcyanobiphenyls **5** are additionally purified by preparative TLC leading to 31–67% isolated yields.

The reaction proved to be compatible with alkyl halides containing different synthetically valuable substituents. This was shown by the example of alkylation of anion **3** arising from the sodium salt of **1^2^**^−^ and benzonitrile **2a** cross-coupling by alkyl bromides **6b**–**f** that were functionalized at their terminal position with a double bond (**6b**) [33], an extra bromine atom (**6c**) [34–35], a cyano (**6d**) or ester group (**6e**), and a 1,3-dioxane fragment (**6f**). The desired 4'-(ω-substituted-alkyl)-biphenyl-4-carbonitriles **5ab**–**af** formed in these reactions in 44–70% yields depending on the nature of the alkylating reagent **6** (Table 1). It should be noted that alkyl halides containing the above-mentioned functional groups have already been used in Birch-type reductive alkylations of polynuclear aromatic compounds [36–39], as well as aromatic acids, and their esters and amides [40–41].

The structures of the obtained products **5ab**–**af** were consistent with the proposed reaction scheme (Scheme 2). It corresponds to the introduction of the cyanophenyl fragment of dianion **1^2^**^−^ into the *para*-position of nitrile **2a** with the formation of cyclohexadienyl anion **3** after rapid decyanation of the primary cross-coupling dianionic product. Next, the alkylation of intermediate **3** occurs at the position *ipso* to the cyano group. This orientation is in agreement with the electronic structure of anion **3**, which is characterized by HOMO localization predominantly at the *ipso* position to the cyano group [24]. This type of HOMO is typical for cyclohexadienyl anions generated from monocyanoarenes [42–43]. The highest yield of **5** (**5ab**, 70%) was obtained for 5-bromopent-1-ene (**6b**). Reactions with ω-substituted alkyl bromides **6d** and **6e** provided lower product yields, which depended on the nature of the alkylating reagent. In the reaction with dibromopentane **6c** we found no disubstitution product – the corresponding 1,5-bis(4-cyanobiphenyl-4-yl)pentane – but the reaction mixture contained traces of compounds with molar masses of 325 and 352 (<1.5% according to GC–MS), which can be attributed to 4'-(5-phenylpentyl)-[1,1'-biphenyl]-4-carbonitrile and 4'-(5-(1-cyanocyclohexa-2,5-dien-1-yl)pentyl)-[1,1'-biphenyl]-4-carbonitrile, respectively. The detection of these byproducts indicates that the bromine-containing product **5ac** is drawn into consecutive reactions with the anionic forms of **2** as an alkylating reagent and that the rate is comparable or higher than the rate of the interaction of dibromide **6c** with anion **3** (compared with [34]). In the case of alkylating reagents **6d**,**e** that contain quite acidic protons in the α-position to the electron-withdrawing substituent, we suppose that partial protonation of the anionic reaction intermediates takes place together with the alkylation, thereby reducing the yield of the target product. The decreased yield in the reaction with acetal **6f** is probably due to the latter’s lower reactivity compared to other alkylating reagents, what was indicated by increased yield of dicyanobiphenyl **4** (14% vs 5% formed in reactions with **6b**–**e**).

To demonstrate the effects of varying the aromatic moiety, a set of aromatic nitriles containing substituents of different natures and positions were used in the reaction (Table 2). We chose the nitriles that had earlier effectively underwent a cross-coupling with dianion **1^2^**^−^ [24–25], in particular, 2- and 3-methyl (**2b**,**c**), 2- and 3-methoxy- (**2d**,**e**), and 2-fluorobenzonitrile (**2f**), as well as 4'-methyl-2-cyanobiphenyl (**2h**). In addition, two new neutral carbonitriles – 2,6-difluorobenzonitrile (**2g**) and 1-cyanonaphthalene (**2i**) – were tested as cross-coupling participants and found to react with dianion **1^2−^**. Alkylation of intermediate anions **3b**–**i** in all these reactions was provided by butyl bromide (**6a**). The composition of the reaction mixtures and the yields of butylcyanobiaryls **5ba**–**ia** were similar to those obtained in the reactions of **1^2^**^−^ with **2a** and alkyl bromides **6a**–**f**. The highest yield (70–75%) was obtained from the reactions with tolunitriles **2b**,**c**. In general, the product yields matched the previously reported [24] dependence of the cross-coupling efficiency on the substituent identity and position in the neutral reaction participant **2**. The reactions of **1^2−^** with 2-substituted benzonitriles provided higher product yields than in the case of the 3-substituted analogs, apparently due to the decrease in spatial hindrance in course of the cross-coupling. The yields depended on the nature of the substituent in **2b**–**f** as follows: yield for methoxy < for fluorine < for methyl. Most likely this order reflects the influence of the substituent electronic effect on the two competing mechanisms within cross-coupling: heterolytic addition of dianion **1^2^**^−^ (S_N_H) to nitrile **2**, and/or electron transfer from **1^2^**^−^ to **2** followed by recombination or relaxation in the primary pair of radical anions **1^·^**^−^ and **2^·^**^−^ (Scheme 2). These probable reaction mechanisms were studied in [23–24] and are beyond consideration of the present paper. In this context, 2,6-difluorobenzonitrile (**2g**) and 4'-methylbiphenyl-2-carbonitrile (**2h**) [25] (which can be considered as benzonitrile, substituted with a *p*-tolyl fragment at the 2 position) show generally the same behavior as all the explored benzonitriles.

**Table 2:** Interaction of dianion **1^2^**^–^ with cyanoarenes **2b**–**i**, followed by alkylation of intermediate anions **3b–i** with butyl bromide **6a**.

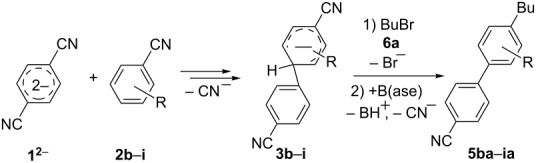				

Entry	Substituted benzonitrile, **2**	Alkyl halide, **6**	Alkylcyanobiphenyl product, **5**	Product yield^a^

1	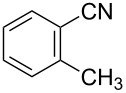 **2b**	BuBr **6a**	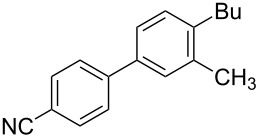 **5ba**	75 (67)
2	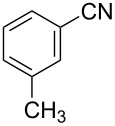 **2c**	BuBr **6a**	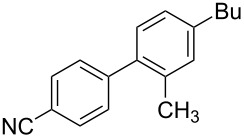 **5ca**	70 (65)
3	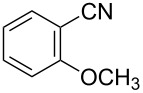 **2d**	BuBr **6a**	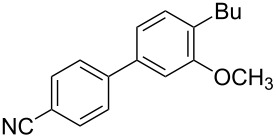 **5da**	66 (56)
4	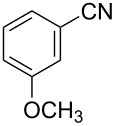 **2e**	BuBr **6a**	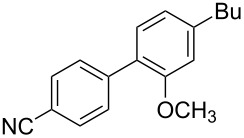 **5ea**	35 (31)
5	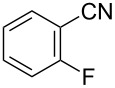 **2f**	BuBr **6a**	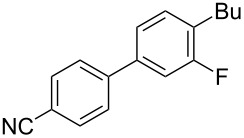 **5fa**	55 (47)
6	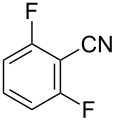 **2g**	BuBr **6a**	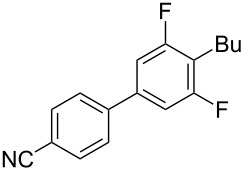 **5ga**	69 (60)
7	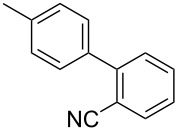 **2h**	BuBr **6a**	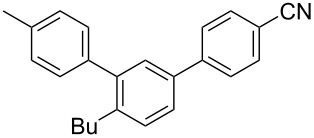 **5ha**	68 (62)
8	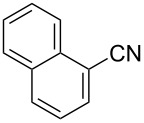 **2i**	BuBr **6a**	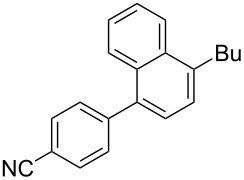 **5ia**	66 (50)

^a^NMR yield, % (isolated yield, %) calculated as mean values of no less than three runs. Deviation does not exceed 5%.

**Scheme 3:**
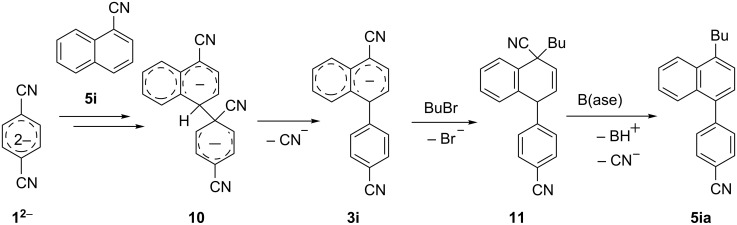
*para*-Cyanophenylation of 1-cyanonaphthalene **5i** by dianion **1^2^**^−^ with subsequent butylation providing 4-(4-butylnaphthalen-1-yl)benzonitrile (**5ia**).

The result obtained in the reaction with 1-naphthonitrile (**2i**) deserves separate discussion because the formation of 4-(4-butylnaphthalen-1-yl)benzonitrile (**5ia**) is the first evidence that dianion **1^2^**^−^ can effectively undergo the cross-coupling with an annulated aromatic nitrile (Table 2, entry 8). The structure of **5ia** shows that a new bond forms between the carbon atoms at position 1 of dianion **1^2^**^−^ and position 4 of **2i**, leading to the formation of the dimeric dianion **10** (Scheme 3). Further transformation of this primary product into butylbiaryl **5ia** is via the pathways described above, i.e., a rapid and irreversible decyanation of dianion **10** with the formation of long-living monoanion **3i**, and the alkylation of anion **3i** at the position *ipso* to the cyano group providing alkyldihydro product **11**, which undergoes fast dehydrocyanation in basic reaction media thus forming final aromatic product **5ia**. The high *ipso*-regioselectivity of the bulylation of monoanion **3i** is typical for an alkylation with primary alkyl halides of cyanocyclohexadienyl anions derived by two-electron reduction of aromatic mononitriles in liquid ammonia, which was studied earlier and was found to proceed via an S_N_2 mechanism [26,35].

## Conclusion

We propose a one-pot method of the transition-metal-free biaryl cross-coupling for the preparation of 4'-alkyl-4-cyanobiaryls as potentially valuable building blocks [3–9,20,44] with a variable structure of aromatic moieties (biphenylic, *m*-terphenylic or phenylnaphthylic), as well as an alkyl moiety. The main advantages of this method are experimental simplicity, utilization of commercially accessible reagents (terephthalonitrile, metallic sodium, substituted aromatic monocarbonitriles, alkyl bromides), together with a recyclable solvent – liquid ammonia – which is now considered as a green solvent [45–47]. Our approach is compatible with a variety of different synthetically valuable substituents both in the aromatic part (methyl and methoxy groups, and fluorine atoms) and the side-chain (double bond, the bromine atom, ester and cyano groups, and 1,3-dioxane fragment). The presence of such substituents in the products opens up the possibility of further synthetic modifications.

## Supporting Information

File 1 Experimental section and ^1^H, ^13^C and ^19^F NMR spectra of all synthesized compounds.
