# Supplementary material for: One-pot synthesis of 4′-alkyl-4-cyanobiaryls on the basis of the terephthalonitrile dianion and neutral aromatic nitrile cross-coupling
Source: Beilstein J Org Chem. 2016 Jul 25;12:1577–84. doi: 10.3762/bjoc.12.153 (PMC4979632; doi:10.3762/bjoc.12.153)

Supporting Information

for

One-pot synthesis of 4′-alkyl-4-cyanobiaryls on the basis of the terephthalonitrile dianion and neutral aromatic nitrile cross-coupling

Roman Yu. Peshkov[1,2], Elena V. Panteleeva*[1,2], Wang Chunyan[2,3], Evgeny V. Tretyakov[1,2] and Vitalij D. Shteingarts[1,§]

Address: [1]Laboratory of the Investigation of Nucleophilic and Radical Ionic Reactions, N.N. Vorozhtsov Novosibirsk Institute of Organic Chemistry of Siberian Branch of Russian Academy of Sciences, Ac. Lavrentiev Avenue, 9, Novosibirsk, 630090, Russia, [2]Natural Sciences Department, Novosibirsk State University, Pirogova St., 2, Novosibirsk, 630090, Russia and [3]Heilongjang University, Xuefu Road, 74, Harbin, 150080, China

Email: Elena V. Panteleeva - pantel@nioch.nsc.ru

*Corresponding author

[§]Deceased

Experimental section and ^{1}H, ^{13}C and ^{19}F NMR spectra of all synthesized compounds

Contents

1. Experimental section s3
2. ^{1}H, ^{13}C and ^{19}F NMR spectra of synthesized compounds. s8
   4'-(Pent-4-en-1-yl)biphenyl-4-carbonitrile, ^{1}H (**5ab**) s8
   4'-(Pent-4-en-1-yl)biphenyl-4-carbonitrile, ^{13}C (**5ab**) s9
   4'-(5-Bromopent-1-yl)biphenyl-4-carbonitrile, ^{1}H (**5ac**) s10
   4'-(5-Bromopent-1-yl)biphenyl-4-carbonitrile, ^{13}C (**5ac**) s11
   4'-(4-Cyanobutyl)biphenyl-4-carbonitrile, ^{1}H (**5ad**) s12
   4'-(4-Cyanobutyl)biphenyl-4-carbonitrile, ^{13}C (**5ad**) s13
   Ethyl 6-(4'-cyanobiphenyl-4-yl)hexanoate, ^{1}H (**5ae**) s14
   Ethyl 6-(4'-cyanobiphenyl-4-yl)hexanoate, ^{13}C (**5ae**) s15
   4'-(2-(1,3-Dioxan-2-yl)ethyl)biphenyl-4-carbonitrile, ^{1}H (**5af**) s16
   4'-(2-(1,3-Dioxan-2-yl)ethyl)biphenyl-4-carbonitrile, ^{13}C (**5af**) s17
   4'-Butyl-3'-methylbiphenyl-4-carbonitrile, ^{1}H (**5ba**) s18
   4'-Butyl-3'-methylbiphenyl-4-carbonitrile, ^{13}C (**5ba**) s19
   4'-Butyl-2'-methylbiphenyl-4-carbonitrile, ^{1}H (**5ca**) s20
   4'-Butyl-2'-methylbiphenyl-4-carbonitrile, ^{13}C (**5ca**) s21
   4'-Butyl-3'-methoxylbiphenyl-4-carbonitrile, ^{1}H (**5da**) s22
   4'-Butyl-3'-methoxylbiphenyl-4-carbonitrile, ^{13}C (**5da**) s23
   4'-Butyl-2'-methoxylbiphenyl-4-carbonitrile, ^{1}H (**5ea**) s24

4'-Butyl-2'-methoxylbiphenyl-4-carbonitrile, ^{13}C (**5ea**)........ s25
4'-Butyl-3'-fluorobiphenyl-4-carbonitrile, ^{1}H (**5fa**) s26
4'-Butyl-3'-fluorobiphenyl-4-carbonitrile, ^{13}C (**5fa**) s27
4'-Butyl-3'-fluorobiphenyl-4-carbonitrile, ^{19}F (**5fa**)........ s28
4'-Butyl-3',5'-difluorobiphenyl-4-carbonitrile, ^{1}H (**5ga**)........ s29
4'-Butyl-3',5'-difluorobiphenyl-4-carbonitrile, ^{13}C (**5ga**)........ s30
4'-Butyl-3',5'-difluorobiphenyl-4-carbonitrile, ^{19}F (**5ga**) s31
4'-Butyl-4"-methyl-[1,1':3',1"-terphenyl]-4-carbonitrile, ^{1}H (**5ha**)........ s32
4'-Butyl-4"-methyl-[1,1':3',1"-terphenyl]-4-carbonitrile, ^{13}C (**5ha**)........ s33
4-Butyl-1-(4-cyanophenyl)naphthalene, ^{1}H (**5ia**)........ s34
4-Butyl-1-(4-cyanophenyl)naphthalene, ^{13}C (**5ia**)........ s35

1. Experimental section

General. ^{1}H, ^{13}C and ^{19}F NMR spectra of all compounds were acquired on a Bruker Avance-III 500 instrument at 500.13 MHz, 125.76 MHz and 470.59 MHz, respectively, in [D$_6$]acetone or [D]chloroform, chemical shifts (δ) of ^{1}H and ^{13}C are in ppm relative to TMS using the solvent signals as the internal standard (δH = 2.05 ppm, δC = 29.8 and 206.3 ppm for [D$_6$]acetone, δH = 7.25 ppm, δC = 77.15 ppm for [D]chloroform), the internal standard for ^{19}F spectra was C_6F_6 (–162.9 ppm). Signal assignment and structure justification were carried out on the HSQC and HMBC data and, when appropriate, on the analysis of C–F spin–spin coupling constants. Numeration of atoms in compounds **5ae, 5af** was changed for more convenient description (see below for details). IR spectra were recorded on a Vector-22 instrument for samples pelleted with KBr (0.25%). The GC–MS analysis was performed on a Hewlett-Packard G1081A instrument consisting of an HP-5890 Series II gas chromatograph and an HP-5971 mass-selective detector (IE, 70 eV) with an HP5 capillary column. The precise molecular ion weights were determined on a DFS instrument. Elemental analysis was carried out in a Carlo Erba automatic C, H, N-analyzer model 1106. Liquid ammonia was purified just before use by dissolving in it metallic sodium, followed by distillation into a reaction vessel, cooled to −80 to −70 °C. Metallic sodium was freed from oxide film under dry hexane. Commercial terephthalonitrile was purified by sublimation. Carbonitriles **2a–i** and alkyl halides **6a–f** were purchased (ABCR, Alfa Aesar or Sigma-Aldrich) and used without further purification. Melting points are uncorrected.

General procedure for the synthesis of alkylcyanobiaryls 5. The first reaction stage was carried out in a similar manner as described earlier:[1] metallic sodium (237 mg, 10.3 mmol) was added to a stirred suspension of dinitrile **1** (640 mg, 5.0 mmol) in liquid ammonia (40–50 mL) at –33 °C under an atmosphere of evaporating ammonia. The mixture was kept for 5 min thus providing a dark-brown suspension of the dianion $\mathbf{1}^{2-}$ salt. Monocarbonitrile **2** (10.0 mmol) was added to a stirred suspension of $\mathbf{1}^{2-}$ salt and the reaction mixture was stirred for 1.5 h at –33 °C, then the alkyl halide **6** (10 mmol) was added and stirring was continued for 1.5 h. Then Et_2O (ca. 30 mL) was added and the reaction mixture was put into contact with air. The stirring was continued until the ammonia was evaporated completely and room temperature was reached. Water (ca. 30 mL) was poured onto the residue to dissolve inorganic salts. The Et_2O layer was separated, the products from the water fraction were extracted with Et_2O (3 × 30 mL). The combined organic extract was washed with brine, dried with $MgSO_4$, the desiccant was filtered off and the solvent was removed. The crude residue was analyzed by ^{1}H NMR spectroscopy and GC–MS. Then the excess of alkyl halide, benzonitrile **2** and partially products **7–9** were distilled under reduced pressure with heating to 70–80°C. The pure products were isolated via preparative TLC on glass plates with a fixed layer of silica gel (60 PF_{254}, Merck) and hexane/Et_2O mixture (10 vol % of Et_2O) as eluent.

4'-(Pent-4-en-1-yl)biphenyl-4-carbonitrile (**5ab**):

Isolated yield 64%. Colorless oil. ^{1}H NMR (500.13 MHz, [D]chloroform): δ = 7.69 (d, J = 8.5 Hz, 2H, 3-H and 5-H), 7.67 (d, J = 8.5 Hz, 2H, 2-H and 6-H), 7.50 (d, J = 8.1 Hz, 2H, 2`-H and 6`-H), 7.30 (d, J = 8.1 Hz, 2H, 3`-H and 5`-H), 5.84 (ddt, J = 17, 10, 6.6 Hz, 1H, 4``-H), 5.04 (dm, J = 17 Hz, 1H, 5``-H), 5.00 (dm, J = 10 Hz, 1H, 5``-H), 2.68 (t, J = 7.8 Hz, 2H, 1``-H), 2.12 (m, 2H, 3``-H), 1.76 (m, 2H, 2``-H) ppm. ^{13}C NMR (125.77 MHz, [D]chloroform): δ = 145.8 (1C, 1-C), 143.5 (1C, 4`-C), 138.6 (1C, 4``-C), 136.8 (1C, 1`-C), 132.7 (2C, 3-C and 5-C), 129.4

1. Peshkov, R. Y.; Panteleeva, E. V.; Shchegoleva, L. N.; Bagryanskaya, I. Y.; Rybalova, T. V.; Vasilieva, N. V.; Shteingarts, V. D. *European J. Org. Chem.*, **2015**, *20,* 4524–4531.

(2C, 3`-C and 5`-C), 127.6 (2C, 2-C and 6-C), 127.3 (2C, 2`-C and 6`-C), 119.2 (1C, CN), 115.1 (1C, 5``-C), 110.8 (1C, 4-C), 35.1 (1C, 1``-C), 33.4 (1C, 3``-C), 30.7 (1C, 2``-C) ppm. UV-Vis (C_2H_5OH), λ_{max}/nm (lg ε): 216 (4.53), 281 (4.69). IR (thin layer): $\tilde{\nu}$ = 2225 (C≡N) cm^{-1}. HRMS: calculated for $C_{18}H_{17}N$ [M^+] 247.1356; found 247.1358. MS (EI), m/z (I_{rel}., %): 247 (6) [M^+], 205 (100), 192 (40), 177 (4).

4'-(5-Bromopent-1-yl)biphenyl-4-carbonitrile (**5ac**):

Isolated yield 55%. Colorless oil. ^{1}H NMR (500.13 MHz, [D]chloroform): δ = 7.68 (d, J = 8.4 Hz, 2H, 3-H and 5-H), 7.65 (d, J = 8.4 Hz, 2H, 2-H and 6-H), 7.49 (d, J = 8.0 Hz, 2H, 2`-H and 6`-H), 7.27 (d, J = 8.0 Hz, 2H, 3`-H and 5`-H), 3.40 (t, J = 6.8 Hz, 2H, 5``-H), 2.67 (t, J = 7.7 Hz, 2H1``-H), 1.89 (m, 2H, 4``-H), 1.65 (m, 2H, 3``-H), 1.49 (m, 2H, 2``-H) ppm. $^{13}$C NMR (125.77 MHz, [D]chloroform): δ = 145.8 (1C, 1-C), 143.3 (1C, 4`-C), 136.9 (1C, 1`-C), 132.8 (2C, 3-C and 5-C), 129.4 (2C, 3`-C and 5`-C), 127.7 (2C, 2-C and 6-C), 127.4.(2C, 2`-C and 6`-C), 119.2 (1C, CN), 111.6 (1C, 4-C), 35.6 (1C, 1``-C), 33.8 (1C, 5``-C), 32.8 (1C ,4``-C), 30.6 (1C, 2``-C), 28.0 (1C, 3``-C) ppm. UV-Vis (C_2H_5OH), λ_{max}/nm (lg ε): 215 (3.97), 280 (4.08). IR (thin layer): $\tilde{\nu}$ = 2225 (C≡N) cm^{-1}. HRMS: calculated for $C_{18}H_{18}BrN$ [M^+] 327.0617; found 327.0616. MS (EI), m/z (I_{rel}., %): 329, 327 (45) [M^+], 192 (100), 165 (10).

4'-(4-Cyanobut-1-yl)biphenyl-4-carbonitrile (**5ad**):

Isolated yield 52%. White solid, m.p. 113.5-115.2°C. ^{1}H NMR (500.13 MHz, [D]chloroform): δ = 7.71 (d, J = 8.4 Hz, 2H, 3-H and 5-H), 7.67 (d, J = 8.4 Hz, 2H, 2-H and 6-H), 7.52 (d, J = 8.1 Hz, 2H, 2`-H and 6`-H), 7.29 (d, J = 8.1 Hz, 2H, 3`-H and 5`-H), 2.72 (t, J = 7.5 Hz, 2H, 1``-H), 2.38 (t, J = 7.0 Hz, 2H, 4``-H), 1.83 (m, 2H, 2``-H), 1.72 (m, 2H, 3``-H) ppm. ^{13}C NMR (125.77 MHz, [D]chloroform): δ = 145.6 (1C, 1-C), 142.2 (1C, 4`-C), 137.3 (1C, 1`-C), 132.8 (2C, 3-C and 5-C), 129.3 (2C, 3`-C and 5`-C), 127.7 (2C, 2-C and 6-C), 127.5 (2C, 2`-C and 6`-C), 119.6 (1C, 4``-C–*CN*), 119.1 (1C, 4C–*CN*), 111.0 (1C, 4-C), 34.9 (1C, 1``C), 30.3 (1C, 3``-C), 25.1 (1C, 2``-C), 17.3 (1C, 4``-C) ppm. UV-Vis (C_2H_5OH), λ_{max}/nm (lg ε): 279 (4.39). IR (KBr): $\tilde{\nu}$ = 2225 (C≡N), 2247 (C≡N) cm^{-1}. HRMS: calculated for $C_{18}H_{16}N_2$ [M^+] 260.1308; found 260.1304. MS (EI), m/z (I_{rel}., %): 260 (50) [M^+], 192 (100), 165 (10). Elemental analysis $C_{18}H_{16}N_2$ (260.34): calcd. C 83.04, H 6.19; N 10.76; found C 82.95, H 6.02, N 11.11.

Ethyl 6-(4'-cyanobiphenyl-4-yl)hexanoate (**5ae**):

Isolated yield 51%. White solid , m.p. 61.9-64.8°C. ^{1}H NMR (500.13 MHz, [D]chloroform): δ = 7.70 (d, J = 8.6 Hz, 2H, 3-H and 5-H), 7.67 (d, J = 8.6 Hz, 2H, 2-H and 6-H), 7.50 (d, J = 8.2 Hz, 2H, 2`-H and 6`- H), 7.28 (d, J = 8.2 Hz, 2H, 3`-H and 5`-H), 4.12 (q, J = 7.1 Hz, 2H, 7``-H), 2.67 (t, J = 7.7 Hz, 2H, 1``-H), 2.30 (t, J = 7.5 Hz, 2H, 5``-H), 1.68 (m, 4H, 2``-H and 4``-H), 1.40 (m, 2H, 3``-H), 1.24 (t, J = 7.1 Hz, 2H, 8``- H) ppm. $^{13}$C NMR (125.77 MHz, [D]chloroform): δ = 173.8 (1C, 6``-C), 145.8 (1C ,1-C), 143.5 (1C, 4`-C), 136.8 (1C, 1`-C), 132.7 (2C, 3-C and 5-C), 129.3 (2C, 3`-C and 5`-C), 127.6 (2C, 2-C and 6-C), 127.2 (2C, 2`-C and 6`-C), 119.1 (1C, CN), 110.1 (1C, 4-C), 60.3 (1C, 7``-C), 35.5 (1C, 1``-C), 34.4 (1C, 5``-C), 31.0 (1C ,2``-C), 28.9 (1C, 3``-C), 24.9 (1C, 4``-C), 14.4 (1C, 8``-C) ppm. UV-Vis (C_2H_5OH), λ_{max}/nm (lg ε): 280 (3.65). IR (KBr): $\tilde{\nu}$ = 1719 (C=O), 2228 (C≡N) cm^{-1}. MS (EI), m/z (I_{rel}., %): 321 (47) [M^+], 275(9), 231 (21),192 (100). HRMS: calculated for $C_{21}H_{23}NO_2$ [M^+] 321.1724; found 321.1723. Elemental analysis $C_{21}H_{23}NO_2$ (321.42): calcd. C 78.47, H 7.21, N 4.36; found C 78.89, H 7.03, N 4.79.

4'-(2-(1,3-dioxane-2-yl)ethyl)biphenyl-4-carbonitrile (**5af**):

Isolated yield 38%. White solid, m.p. 149.6-153.8 °C. ^{1}H NMR (500.13 MHz, [D]chloroform): δ = 7.70 (d, J = 8.2 Hz, 2H, 3-H and 5-H), 7.66 (d, J = 8.2 Hz, 2H, 2-H and 6-H), 7.50 (d, J = 8.0 Hz, 2H, 2`-H and 6`-H), 7.29 (d, J = 8.0 Hz, 2H, 3`-H and 5`-H), 4.54 (t, J = 5.1 Hz, 1H, 2```-C), 4.12 (m, 2H, 4```-H and 6```-H), 3.76 (m, 2H, 4```-H and 6```-H), 2.77 (t, J = 8.0 Hz, 2H, 1``-H) 2.03-2.15 (m, 1H, 5```-H), 1.90-1.95 (m, 2H, 2``-H), 1.35 (m, 1H, 5```-H) ppm. ^{13}C NMR (125.77 MHz, [D]chloroform): δ = 145.8 (1C, 1-C), 142.9 (1C, 4`-C), 136.9 (1C, 1`-C), 132.7 (2C, 3-C and 5-C), 129.4 (2C, 3`-C and 5`-C), 127.7 (2C, 2-C and 6-C), 127.3 (2C, 2`-C and 6`-C) 119.1 (1C, CN), 110.9 (1C, 4-C), 101.5 (1C, 2```-C), 67.1 (1C, 4```-C and 6```-C), 36.7 (1C, 2``-C), 29.9 (1C, 1``-C), 26.0 (1C, 5```-C) ppm. UV-Vis (C_2H_5OH), λ_{max}/nm (lg ε): 280 (4.54). IR (KBr): $\tilde{\nu}$ = 2224 (C≡N) cm^{-1}. MS (EI), m/z ($I_{rel.}$, %): 293 (6) [M^+], 234 (23), 192 (32), 114 (79), 87 (100). HRMS: calculated for $C_{19}H_{19}NO_2$ [M^+] 293.1410; found 293.1329. Elemental analysis $C_{19}H_{19}NO_2$ (293.37): calcd. C 77.79, H 6.53, N 4.77; found C 77.39, H 6.24, N 4.92.

4'-Butyl-3'-methylbiphenyl-4-carbonitrile (**5ba**):

Isolated yield 67%. Yellowish oil. ^{1}H NMR (500.13 MHz, [D_6]acetone): δ = 7.80 (d, J = 8.7 Hz, 2H, 2`-H and 6`-H), 7.78 (d, J = 8.7 Hz, 2H, 3`-H and 5`-H), 7.47 (d, J = 2.1 Hz, 1H, 2-H), 7.44 (dd, J = 7.8, 2.1 Hz, 1H, 6-H), 7.25 (d, J = 7.8 Hz, 1H, 5-H), 2.65 (t, J = 7.8 Hz, 2H, 1``-H), 2.37 (s, 3H, 3-C–*CH*$_3$), 1.57 (m, 2H, 2``-H), 1.42 (m, J = 7.4 Hz x 5, 2H, 3``-H), 0.95 (t, J = 7.3 Hz, 3H, 4``-H) ppm. ^{13}C NMR (125.76 MHz, [D_6]acetone): δ = 146.2 (1C, 1`-C), 142.5 (1C, 4-C), 137.3 (1C, 3-C), 137.1 (1C, 1-C), 133.2 (2C, 3`-C and 5`-C), 130.3 (1C, 5-C), 129.5 (1C, 2-C), 128.1 (2C, 2`-C and 6`-C), 125.3 (1C, 6-C), 119.3 (1C, C≡N), 111.2 (1C ,4`-C), 33.2 (1C, 1``-C), 33.1 (1C, 2``-C), 23.2 (1C, 3``-C), 19.4 (1C, 3-C-*CH*$_3$), 14.2 (1C, 4``-C) ppm. IR (KBr): $\tilde{\nu}$ = 2226 (C≡N) cm^{-1}. MS (EI), m/z ($I_{rel.}$, %): 249 (37) [M^+], 206 (100), 190 (22). HRMS: calculated for $C_{18}H_{19}N$ [M^+] 249.1512; found 249.1510. Elemental analysis $C_{18}H_{19}N$ (249.36): calcd. C 86.70, H 7.68, N 5.62; found C 85.90, H 8.36, N 5.92.

4'-Butyl-2'-methylbiphenyl-4-carbonitrile (**5ca**):

Isolated yield 65%. Pale yellow solid. ^{1}H NMR (500.13 MHz, [D_6]acetone): δ = 7.82 (d, J = 8.5 Hz, 2H, 3`-H and 5`-H), 7.55 (d, J = 8.5 Hz, 2H, 2`-H and 6`-H), 7.17 (br.m, 1H, 3-H), 7.15 (d, J = 7.7 Hz, 1H, 6-H), 7.12 (dd, J = 7.9, 1.4 Hz, 1H, 5-H), 2.63 (t, J = 7.8 Hz, 2H, 1``-H), 2.24 (s, 3H, 3-C–*CH*$_3$), 1.63 (m, 2H, 2``-H), 1.39 (m, 2H, 3``-H), 0.94 (t, J = 7.4 Hz, 3H, 4``-H) ppm. ^{13}C NMR (125.76 MHz, [D_6]acetone): δ = 147.7 (1C, 1`-C), 143.8 (1C, 4-C), 138.4 (1C, 1-C), 135.6 (1C, 2-C), 132.9 (2C, 3`-C and 5`-C), 131.6 (1C, 3-C), 131.1 (2C, 2`-C and 6`-C), 130.3 (1C, 6-C), 127.1 (1C, 5-C), 119.4 (1C, CN), 111.4 (1C, 4`-C), 35.9 (1C, 1``-C), 34.4 (1C, 2``-C), 23.1 (1C, 3``-C), 20.4 (1C, 3-C–*CH*$_3$), 14.2 (1C, 4``-C) ppm. IR (KBr): $\tilde{\nu}$ = 2226 (C≡N) cm^{-1}. MS (EI), m/z ($I_{rel.}$, %): 249 (48) [M^+], 206 (100), 190 (30). HRMS: calculated for $C_{18}H_{19}N$ [M^+] 249.1512; found 249.1508.

4'-Butyl-3'-methoxybiphenyl-4-carbonitrile (**5da**):

Isolated yield 56%. Yellowish oil. ^{1}H NMR (500.13 MHz, [D_6]acetone): δ = 7.88 (d, J = 8.4 Hz, 2H, 2`-H and 6`-H), 7.83 (d, J = 8.4 Hz, 2H, 3`-H and 5`-H), 7.28 (d, J = 1.6 Hz, 1H, 2-H), 7.26 (d, J = 7.7 Hz, 1H, 5-H), 7.23 (dd, J = 7.7 Hz, J = 1.6 Hz, 1H, 6-H), 3.94 (s, 3H, OCH_3), 2.65 (t, J = 7.7 Hz, 2H, 1``-H), 1.58 (m, 2H, 2``H), 1.37 (m, J = 7.7 Hz x 5, 2H, 3``-H), 0.93 (t, J = 7.5 Hz, 3H, 4``-H) ppm. ^{13}C NMR (125.76 MHz, [D_6]acetone): δ = 159.0 (3-C), 146.5 (1`-C), 138.8 (1-C), 133.4 (2C, 3`-C and 5`-C), 132.6 (4-C), 131.2 (5-C), 128.5 (2C, 2`-C and 6`-C), 120.0 (6-C), 119.5 (CN), 111.5 (4`-C), 110.1 (2-C), 55.9 (OCH_3), 32.8 (2``-C), 30.3 (1``-C), 23.3 (3``-C), 14.2 (4``-C) ppm. IR (KBr): $\tilde{\nu}$ = 2226 (C≡N) cm^{-1}. MS (EI), m/z ($I_{rel.}$, %): 265 (39) [M^+], 222

(100), 192 (39), 190 (17), 165 (17). HRMS: calculated for $C_{18}H_{19}NO$ [M^+] 265.1461; found 265.1465. Elemental analysis $C_{18}H_{19}NO$ (265.36): calcd. C 81.47, H 7.22, N 5.28; found C 81.47, H 7.46, N 5.46.

4'-Butyl-2'-methoxybiphenyl-4-carbonitrile (**5ea**):

Isolated yield 31%. Yellowish oil. ^1H NMR (500.13 MHz, [D_6]acetone): δ = 7.77 (d, J = 8.5 Hz, 2H, 3`-H and 5`-H), 7.72 (d, J = 8.5 Hz, 2H, 2`-H and 6`-H), 7.26 (d, J = 7.7 Hz, 1H, 6-H), 7.00 (d, J = 1.2 Hz, 1H, 3-H), 6.91 (dd, J = 7.7 Hz, J = 1.2 Hz, 1H, 5-H), 3.83 (s, 3H, OCH_3), 2.66 (t, J = 7.8 Hz, 2H, 1``-H), 1.64 (m, 2H, 2``H), 1.38 (m, J = 7.5 Hz x5, 2H, 3``-H), 0.93 (t, J = 7.4 Hz, 3H, 4``-H) ppm. ^{13}C NMR (125.76 MHz, [D_6]acetone): δ = 157.4 (1C, 2-C), 146.2 (1C, 4-C), 144.5 (1C, 1`-C), 132.5 (2C, 3`-C and 5`-C), 131.1(4) (1C, 6-C), 131.1(0) (2C, 2`-C and 6`-C), 126.7 (1C, 1-C), 121.9 (1C, 5-C), 119.6 (1C, CN), 112.8 (1C, 3-C), 110.8 (1C, 4`-C), 55.9 (1C, OCH_3), 36.3 (1C, 1``-C), 34.3 (1C, 2``-C), 23.0 (1C, 3``-C), 14.2 (1C, 4``-C) ppm. IR (KBr): $\tilde{\nu}$ = 2226 (C≡N) cm^{-1}. MS (EI), m/z ($I_{rel.}$, %): 265 (93) [M^+], 223 (100), 208 (15), 206 (24), 190 (22), 178 (12). HRMS: calculated for $C_{18}H_{19}NO$ [M^+] 265.1461; found 265.1458. Elemental analysis $C_{18}H_{19}NO$ (265.36): calcd. C 81.47, H 7.22, N 5.28; found C 81.43, H 8.16, N 5.34.

4'-Butyl-3'-fluorobiphenyl-4-carbonitrile (**5fa**):

Isolated yield 47%. Yellowish oil. ^1H NMR (500.13 MHz, [D_6]acetone): δ = 7.89 (d, J = 8.7 Hz, 2H, 2`-H and 6`-H), 7.85 (d, J = 8.7 Hz, 2H, 3`-H and 5`-H), 7.50 (dd, J = 7.9, 1.9 Hz, 1H, 6-H), 7.46 (dd, J = 11.3, 1.8 Hz, 1H, 2-H), 7.41 (t, J = 7.9 Hz, 1H, 5-H), 2.70 (t, J = 7.6 Hz, 2H, 1``-H), 1.63 (m, 2H, 2``-H), 1.39 (m, 2H, 3``-H), 0.94 (t, J = 7.4 Hz, 3H, 4``-H) ppm. ^{13}C NMR (125.76 MHz, [D_6]acetone): δ = 162.4 (d, J_F = 244 Hz, 1C, 3-C), 144.8 (d, J = 2.2 Hz, 1C, 1`-C), 139.6 (d, J = 8.0 Hz, 1C 1-C), 133.6 (s, 2C, 3`-C and 5`-C), 132.4 (d, J = 5.8 Hz, 1C, 5-C), 130.8 (d, J = 16.4 Hz, 1C, 4-C), 128.5 (2C, 2`-C and 6`-C), 123.7 (d, J = 3.2 Hz, 1C, 6-C), 119.3 (1C, CN), 114.6 (d, J = 24.1 Hz, 1C, 2-C), 112.1 (1C, 4`-C), 33.0 (1C, 2``-C), 28.9 (1C, 1``-C), 23.0 (1C, 3``-C), 14.1 (1C, 4``-C) ppm. ^{19}F NMR (470.59 MHz, [D_6]acetone): δ = 45.0 (dd, J = 11.1, 8.1 Hz, 1F, 3-F) ppm. IR (KBr): $\tilde{\nu}$ = 2227 (C≡N) cm^{-1}. MS (EI), m/z ($I_{rel.}$, %): 253 (35) [M^+], 210 (100). HRMS: calculated for $C_{17}H_{16}FN$ [M^+] 253.1261; found 253.1263.

4'-Butyl-3',5'-difluorobiphenyl-4-carbonitrile (**5ga**):

Isolated yield 60%. Yellowish oil, slowly crystallizes in cold. M.p.73°C. ^1H NMR (500.13 MHz, [D_6]acetone): δ = 7.90 (d, J = 8.5 Hz, 2H, 2`-H and 6`-H), 7.86 (d, J = 8.5 Hz, 2H, 3`-H and 5`-H), 7.36 (d, J = 8.6 Hz, 2H, 2-H and 6-H), 2.70 (t, J = 7.6 Hz, 2H, 1``-H), 1.58 (m, 2H, 2``-H), 1.38 (m, J = 7.6 Hz x 5, 2H, 3``-H), 0.93 (t, J = 7.5 Hz, 3H, 4``-H) ppm. ^{13}C NMR (125.76 MHz, [D_6]acetone): δ = 162.6 (dd, J_F=247, 10.0 Hz, 2C, 3-C and 5-C), 143.3 (t, J_F=2.6 Hz, 1C, 1`-C), 140.0 (t, $J_F$=10.1 Hz, 1C, 1-C), 133.7 (s, 2C, 3`-C and 5`-C), 128.6 (s, 2C, 2`-C and 6`-C), 119.1 (s, 1C, C≡N), 119.0 (t, $J_F$=21.0 Hz, 4-C), 112.7 (s, 1C, 4`-C), 110.7 (dd, J_F=20.8, 7.4 Hz, 2C, 2-C and 6-C), 32.4 (s, 1C, 2``-C), 23.0 (s, 1C, 3``-C), 22.5 (t, J_F=2.1 Hz, 1``-C), 14.0 (s, 1C, 4``-C) ppm. ^{19}F NMR (470.59 MHz, [D_6]acetone): δ = 48.2 (d, J =8.0 Hz, 2F, 3-F and 5-F) ppm. IR (KBr): $\tilde{\nu}$ = 2227 (C≡N) cm^{-1}. MS (EI), m/z ($I_{rel.}$, %): 271 (24) [M^+], 240 (17), 228 (100). HRMS: calculated for $C_{17}H_{15}F_2N$ [M^+] 271.1167; found 271.1166. Elemental analysis $C_{17}H_{15}F_2N$ (271.31): calcd. C 75.26, H 5.57, F 14.00, N 5.16; found C 75.63, H 5.97, F 13.61, N 5.19.

4'-Butyl-4"-methyl-[1,1':3',1"-terphenyl]-4-carbonitrile (**5ha**):

Isolated yield 62%. Yellowish oil. ^1H NMR (500.13 MHz, [D_6]acetone): δ = 7.90 (d, J = 7.6 Hz, 2H, 2-H and 6-H), 7.83 (d, J = 7.6 Hz, 2H, 3-H and 5-H), 7.66 (dd, J = 8.0, 2.0 Hz, 1H, 2`-H),

7.51 (d, J = 2.0 Hz, 1H, 6`-H), 7.45 (d, J = 8.0 Hz, 1H, 3`-H), 7.25 (br. s, 4H, 2``-H, 3``-H, 5``-H and 6``-H), 2.65 (t, J = 7.8 Hz, 2H, α-H), 2.39 (s, 3H, 4``-$CH_3$), 1.48 (m, 2H, β-H), 1.23 (m, J = 7.5 Hz x5, 2H, γ-H), 0.80 (t, J = 7.4 Hz, 2H, δ-H) ppm. $^{13}$C NMR (125.76 MHz, [$D_6$]acetone): δ = 146.0 (1C, 1-C), 143.5 (1C, 3`-C), 141.9 (1C, 4`-C), 139.5 (1C, 1``-C), 137.5 (1C, 4``-C), 137.2 (1C, 1`-C), 133.5 (2C, 3-C and 5-C), 131.1 (1C, 5`-C), 130.0 (2C, 2``-C and 6``-C), 129.7 (2C, 3``-C and 5``-C), 129.5 (1C, 2`-C), 128.5 (2C, 2-C and 6-C), 126.7 (1C, 6`-C), 119.4 (1C, CN), 111.6 (1C, 4-C), 34.2 (1C, β-C), 33.1 (1C, α-C), 23.2 (1C, γ-C), 21.2 (1C, 4``-C-*CH_3*), 14.1 (1C, δ-C) ppm. IR (KBr): $\tilde{\nu}$ = 2226 (C≡N) cm^{-1}. MS (EI), m/z ($I_{rel.}$, %): 325 (54) [M^+], 282 (100), 267 (28). HRMS: calculated for $C_{24}H_{23}N$ [M^+] 325.1825; found 325.1827. Elemental analysis $C_{24}H_{23}N$ (325.46): calcd. C 88.57, H 7.12, N 4.30; found C88.47, H 7.58, N 4.30.8.47, H 7.58, N 4.30.

4-Butyl-1-(4-cyanophenyl)naphthalene (**5ia**):

Isolated yield 50%. While solid. M.p. 68.6°C (diethyl ether). ^{1}H NMR (500.13 MHz, [D_6]acetone): δ = 8.21 (ddd, J = 8.5, 1.3, 0.7 Hz, 1H, 6-H), 7.91 (d, J = 8.6 Hz, 2H, 3`-H and 5`-H), 7.81 (ddd, J = 8.5, 1.3, 0.7 Hz, 1H, 9-H), 7.68 (d, J = 8.6 Hz, 2H, 2`-H and 6`-H) 7.59 (ddd, J = 8.6, 6.8, 1.3 Hz, 1H, 7-H), 7.49 (ddd, J = 8.6, 6.8, 1.3 Hz, 1H, 8-H), 7.46 (d, J = 7.2 Hz, 1H, 3-H), 7.38 (d, J = 7.2, 1H, 2-H), 3.16 (t, J = 7.9 Hz, 2H, 1``-H), 1.77 (m, 2H, 2``-H), 1.50 (m, J = 7.5 Hz x5, 2H, 3``-H), 0.99 (t, J = 7.4 Hz, 3H, 4``-H) ppm. ^{13}C NMR (125.76 MHz, [D_6]acetone): δ = 146.8 (1C, 1`-C), 140.6 (1C, 4-C), 137.6 (1C, 1-C), 133.2 (1C, 5-C), 133.1 (2C, 3`-C and 5`-C), 132.3 (1C, 10-C), 131.9 (1C, 2`-C and 6`-C), 127.7 (1C, 2-C), 127.0 (1C, 8-C), 126.9 (1C, 7-C), 126.8 (1C, 9-C), 126.4 (1C, 3-C), 125.3 (1C, 6-C), 119.4 (1C, CN), 111.9 (1C, 4`-C), 33.9 (1C, 2``-C), 33.5 (1C, 1``-C), 23.5 (1C, 3``-C), 14.3 (1C, 4``-C) ppm. IR (KBr): $\tilde{\nu}$ = 2222 (C≡N) cm^{-1}. MS (EI), m/z ($I_{rel.}$, %): 285 (50) [M^+], 242 (100), 227 (19). HRMS: calculated for $C_{21}H_{19}N$ [M^+] 285.1512; found 285.1514. Elemental analysis $C_{21}H_{19}N$ (285.39): calcd. C 88.38, H 6.71, N 4.91; found C 88.02, H 6.71, N 4.90.

2. ^{1}H, ^{13}C and ^{19}F NMR spectra of synthesized compounds.

4'-(Pent-4-en-1-yl)biphenyl-4-carbonitrile, ^{1}H (5ab)

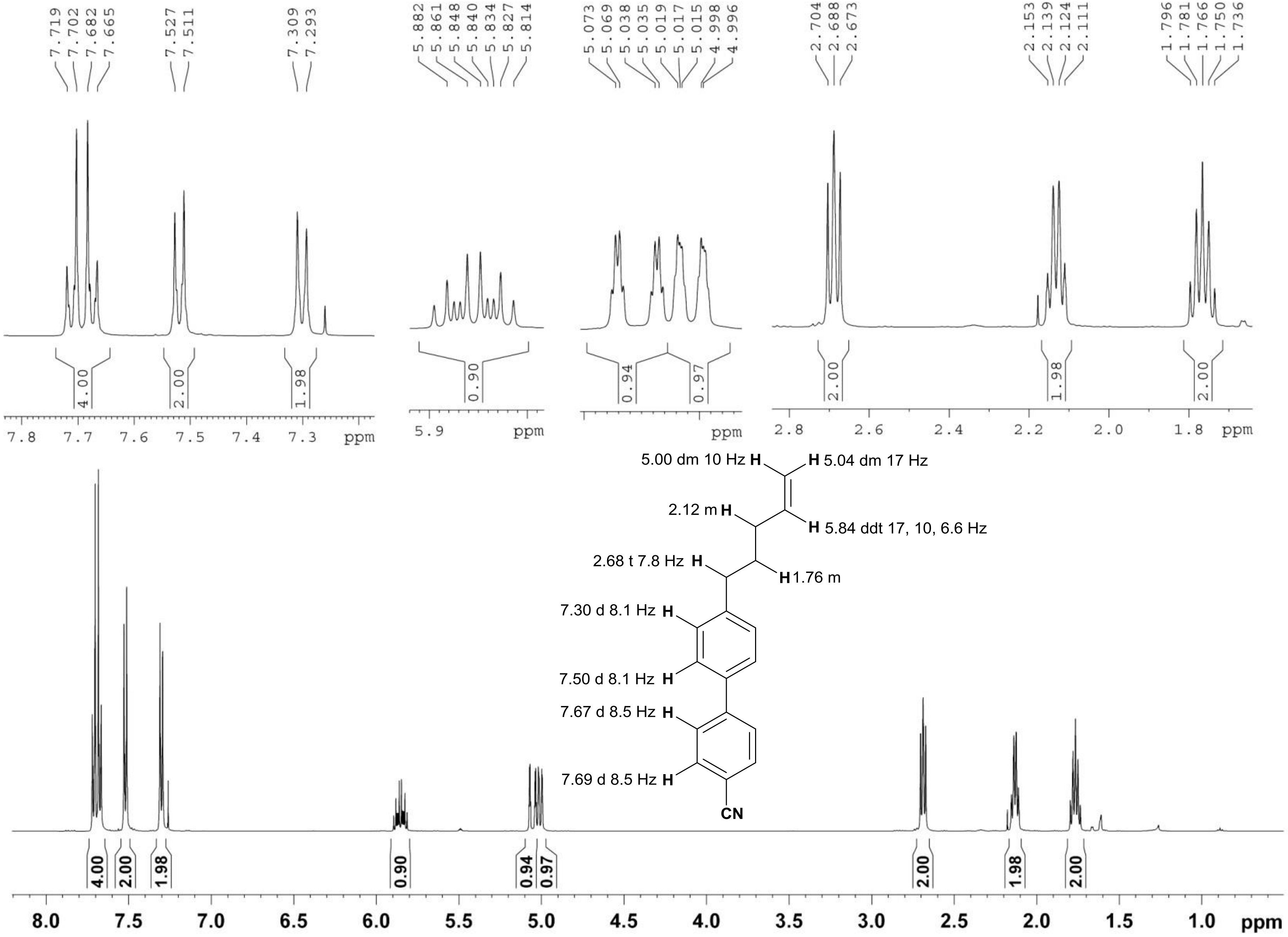

4'-(Pent-4-en-1-yl)biphenyl-4-carbonitrile, ^{13}C (5ab)

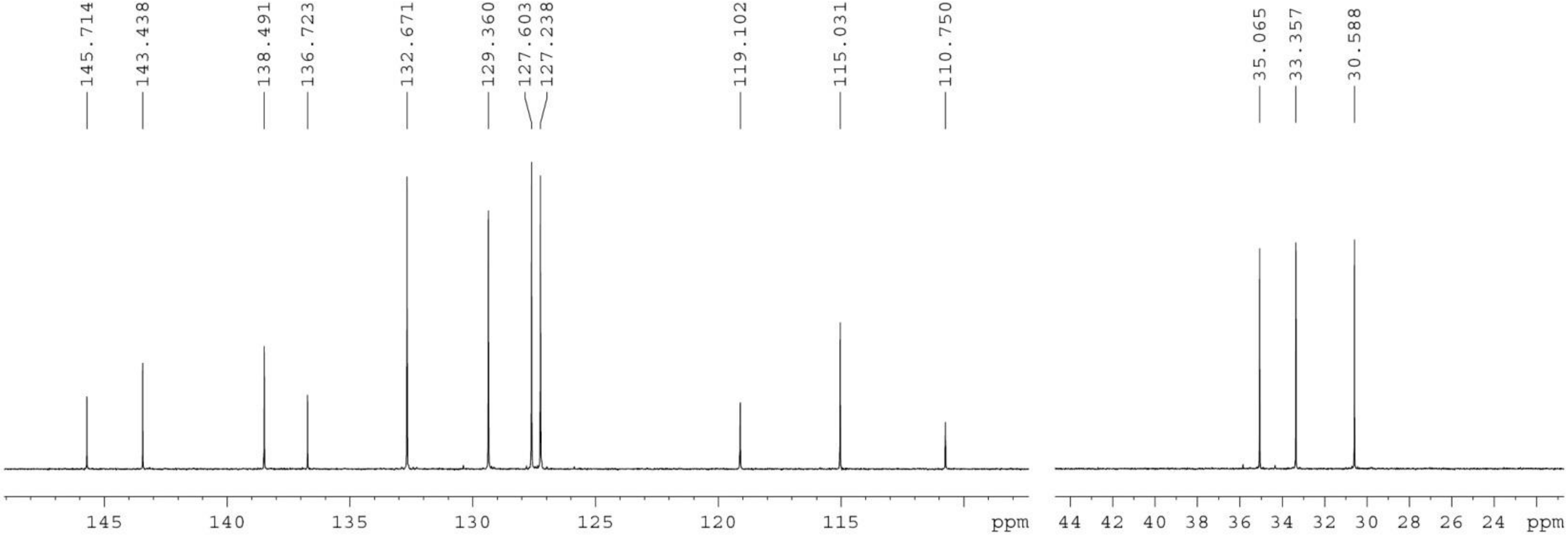

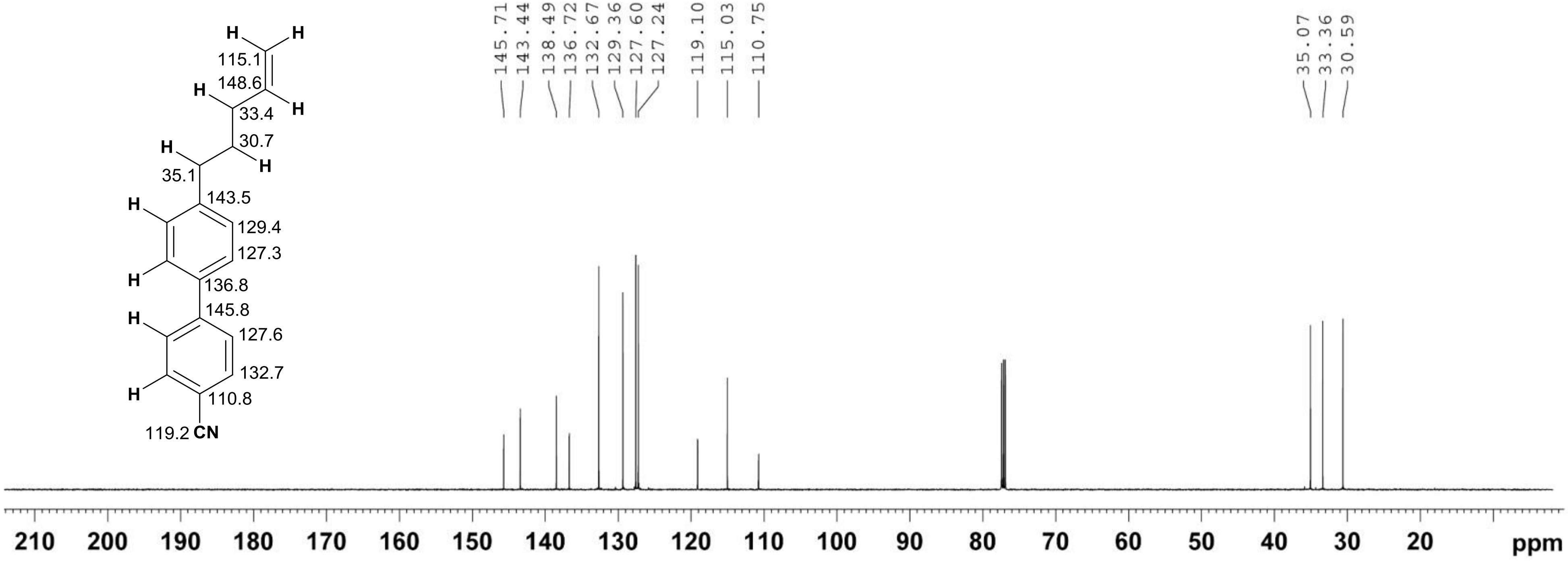

4'-(5-Bromopent-1-yl)biphenyl-4-carbonitrile, ^{1}H (5ac)

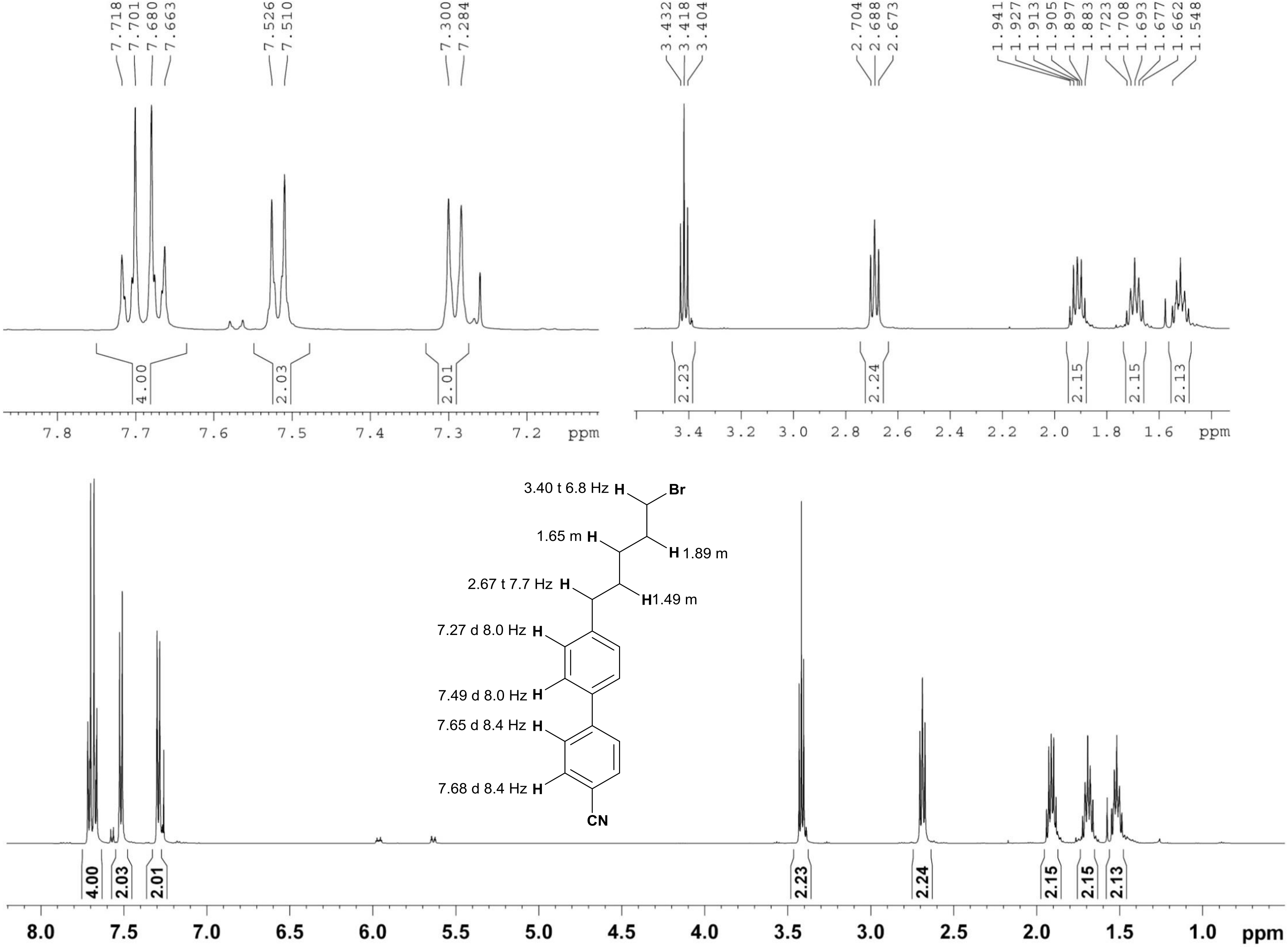

4'-(5-Bromopent-1-yl)biphenyl-4-carbonitrile, ^{13}C (5ac)

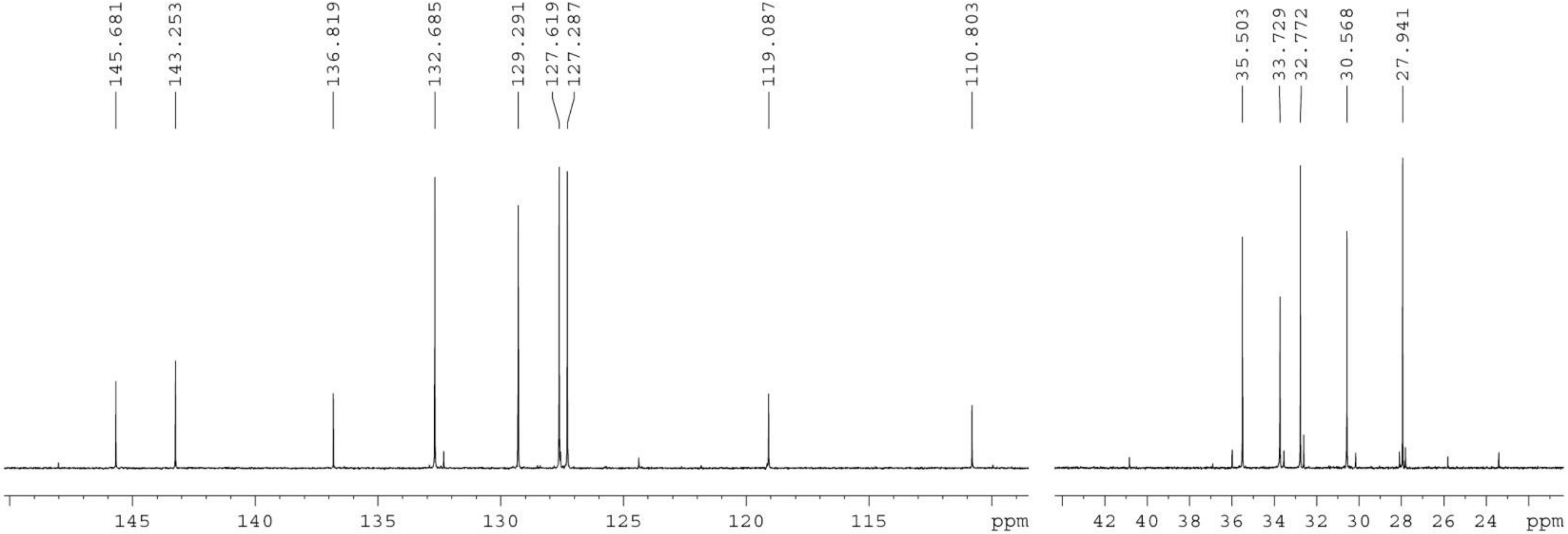

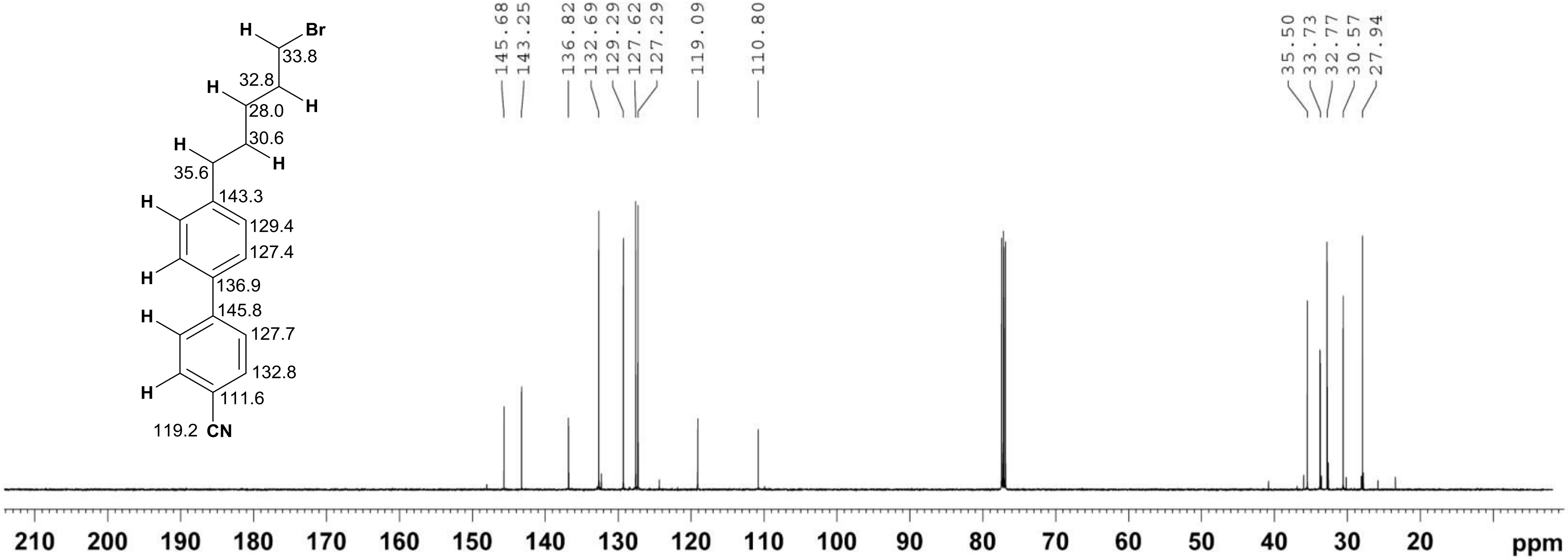

4'-(4-Cyanobutyl)biphenyl-4-carbonitrile, ^{1}H (5ad)

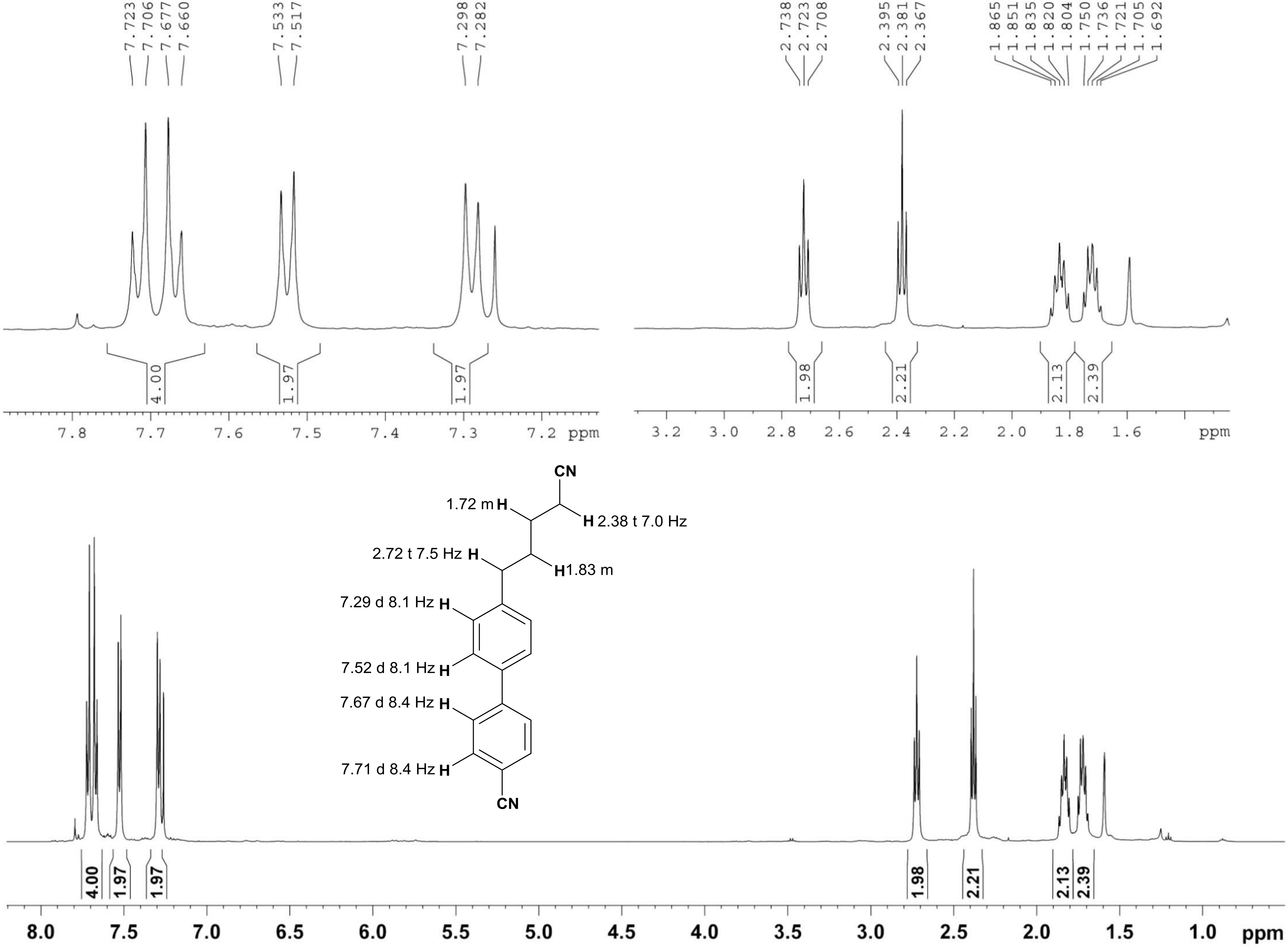

4'-(4-Cyanobutyl)biphenyl-4-carbonitrile, ^{13}C (5ad)

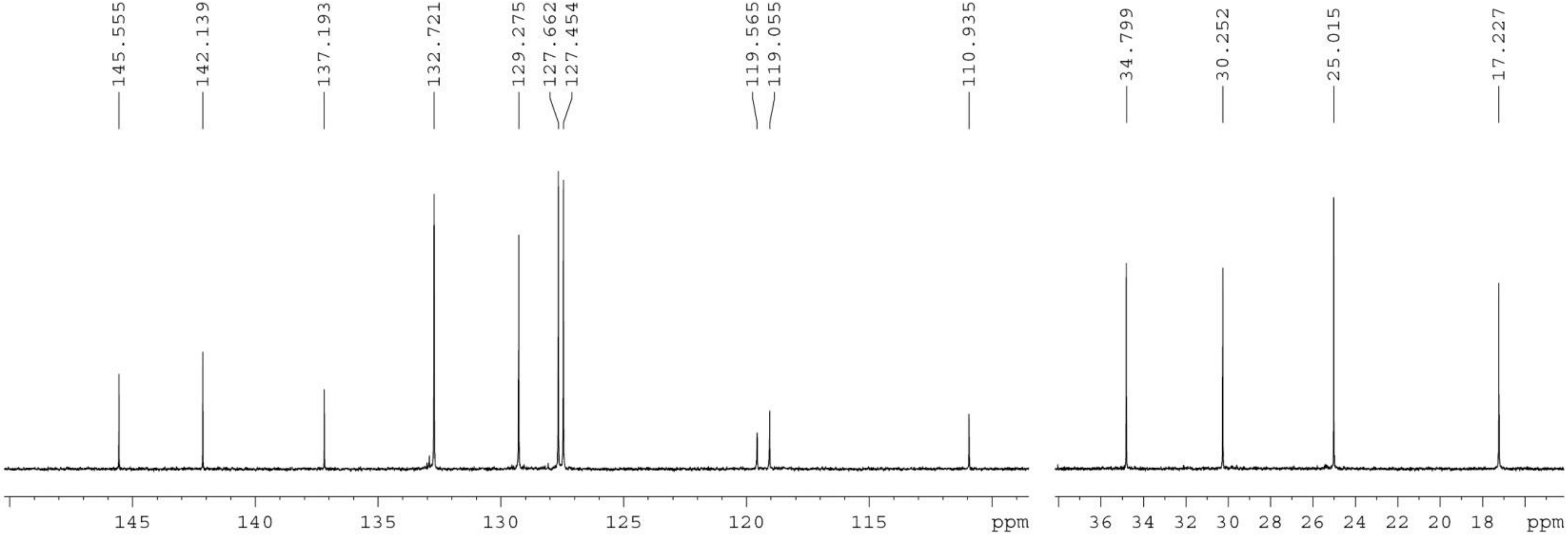

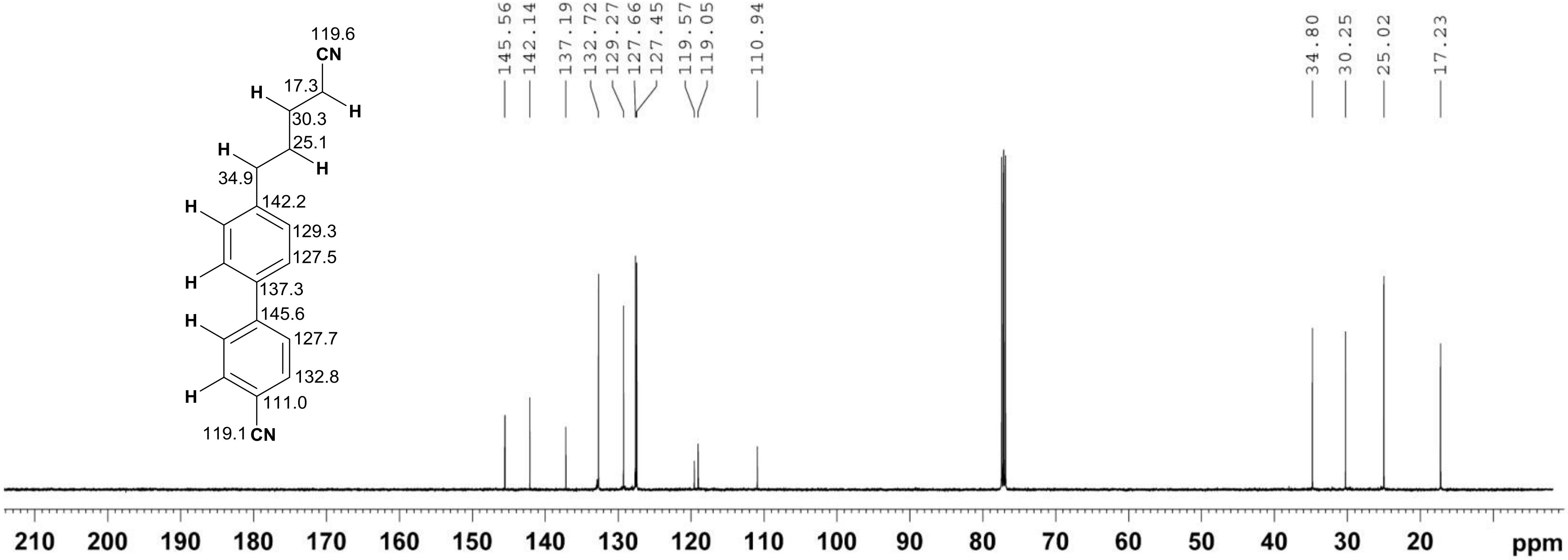

Ethyl 6-(4'-cyanobiphenyl-4-yl)hexanoate, ^{1}H (5ae)

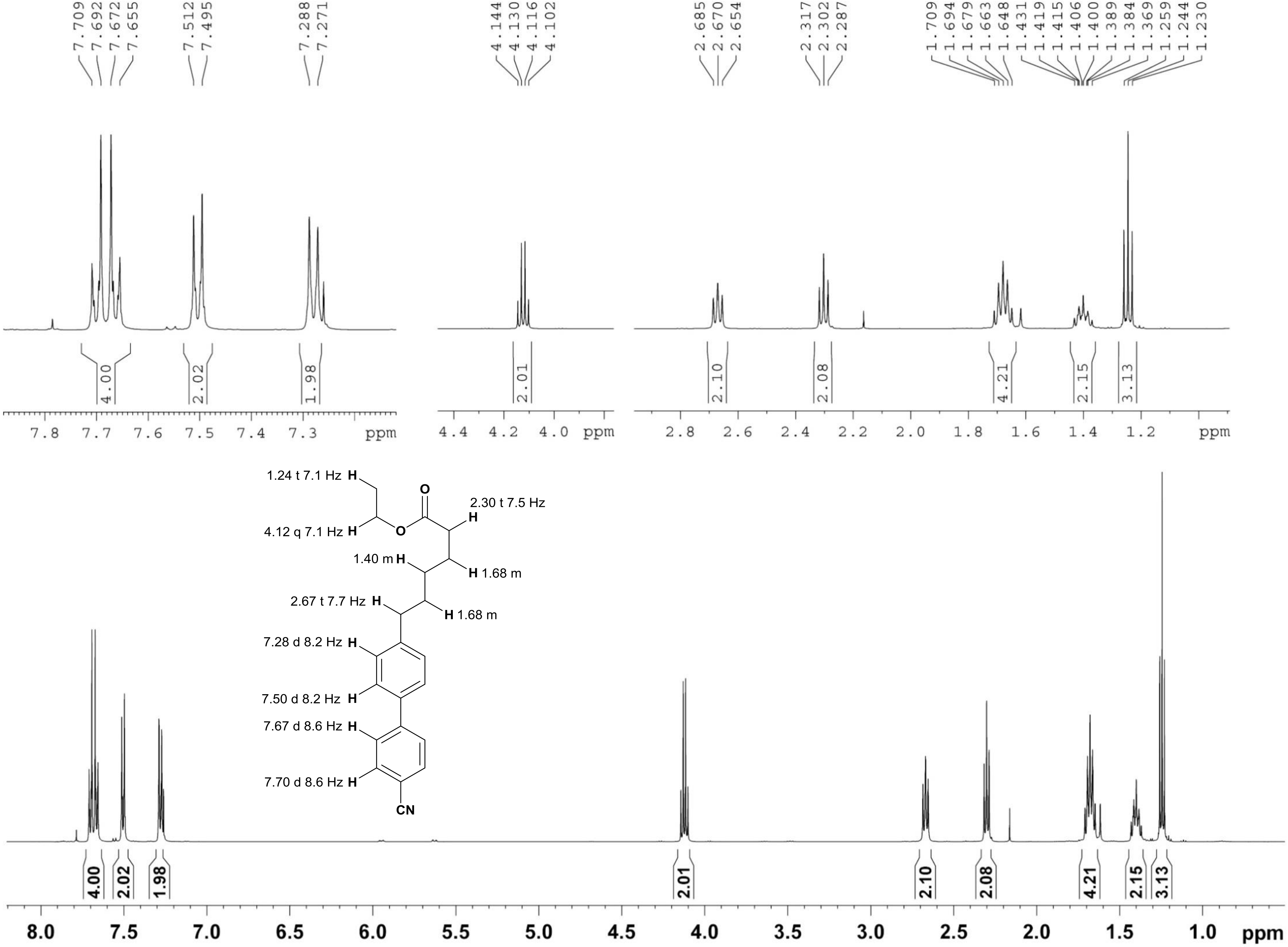

Ethyl 6-(4'-cyanobiphenyl-4-yl)hexanoate, ^{13}C (5ae)

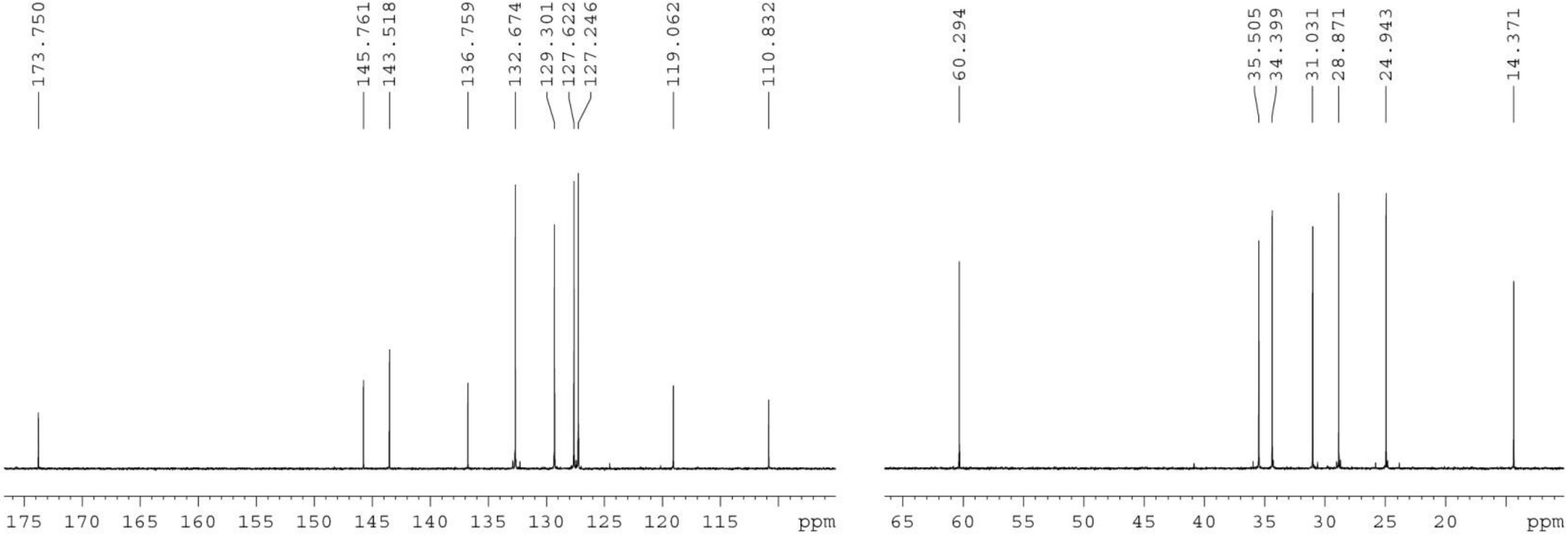

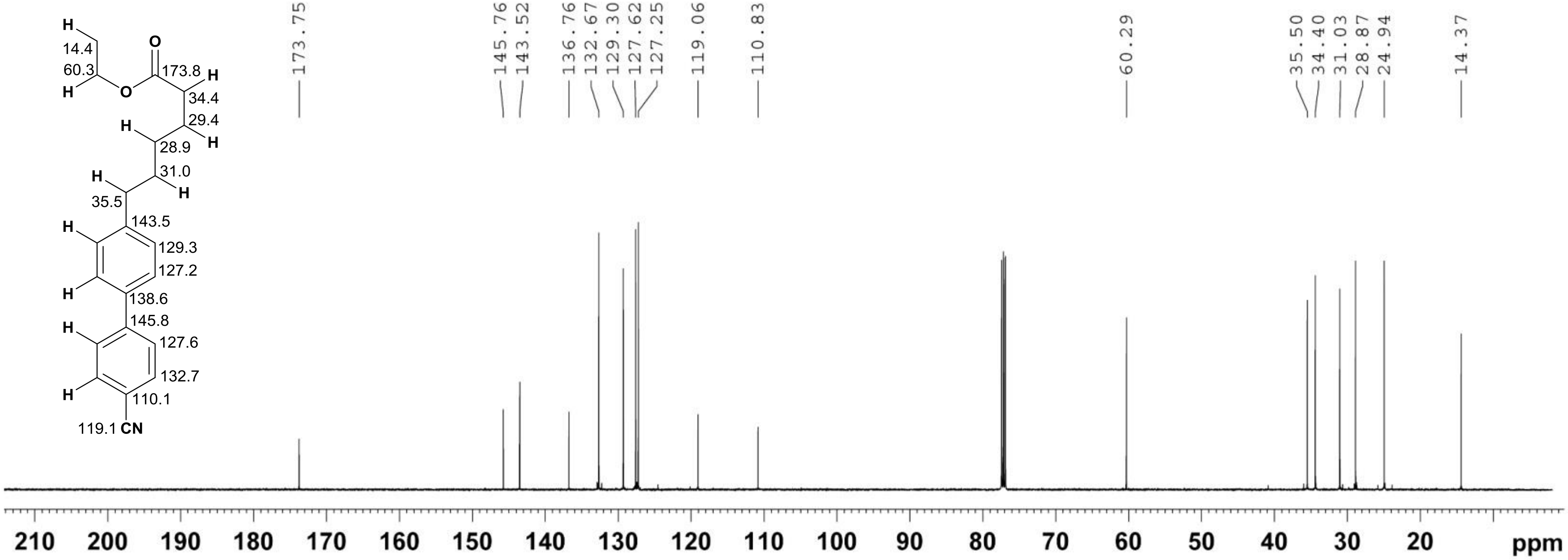

4'-(2-(1,3-Dioxan-2-yl)ethyl)biphenyl-4-carbonitrile, ^{1}H (5af)

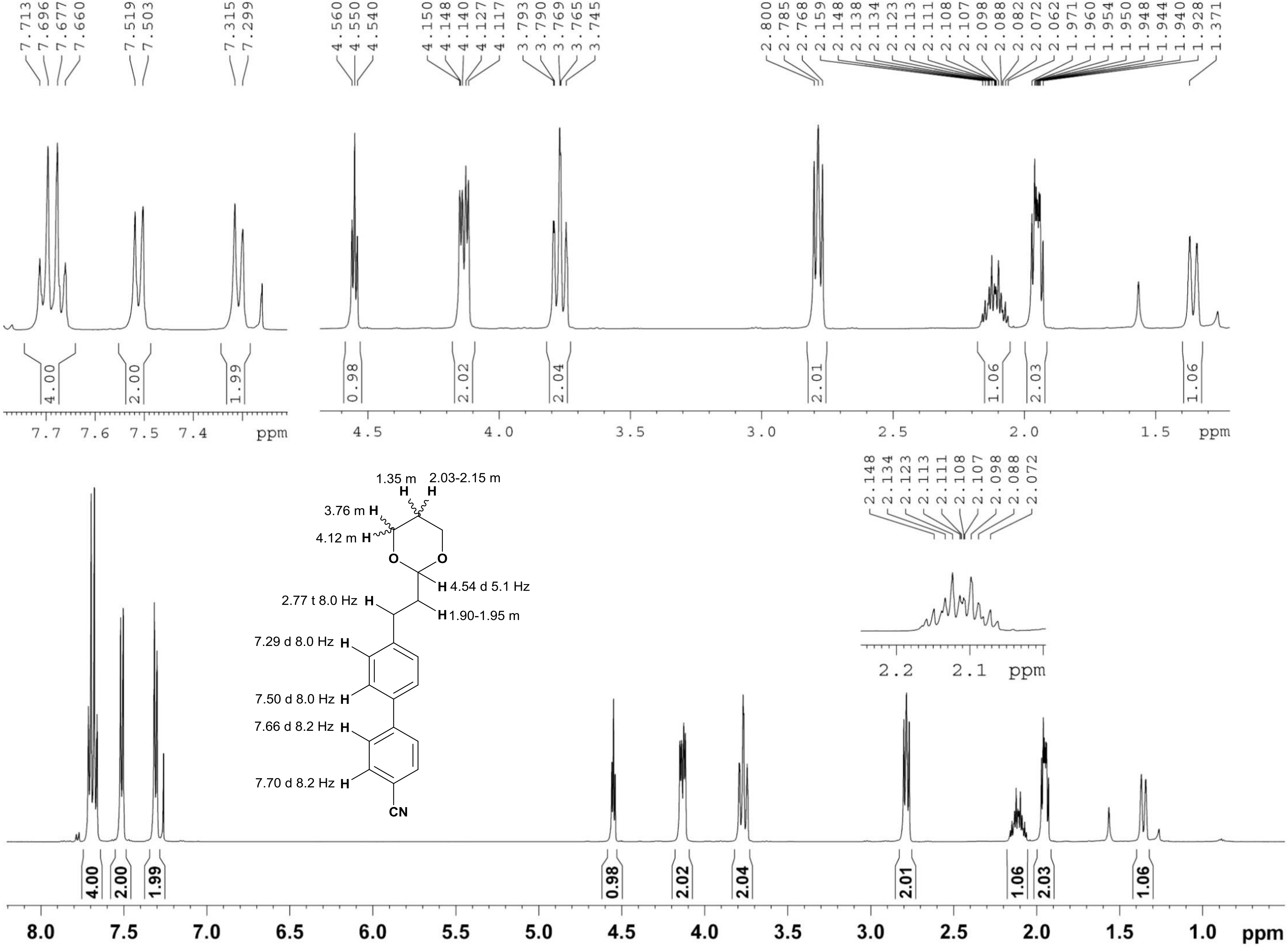

4'-(2-(1,3-Dioxan-2-yl)ethyl)biphenyl-4-carbonitrile, ^{13}C (5af)

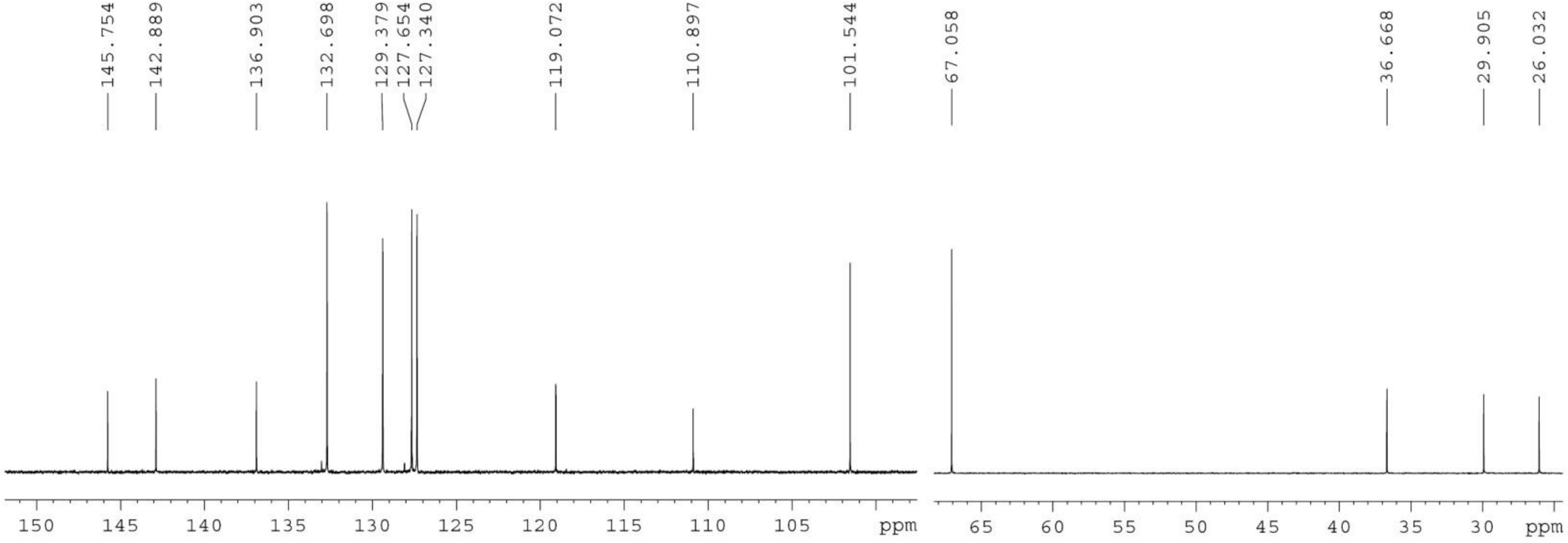

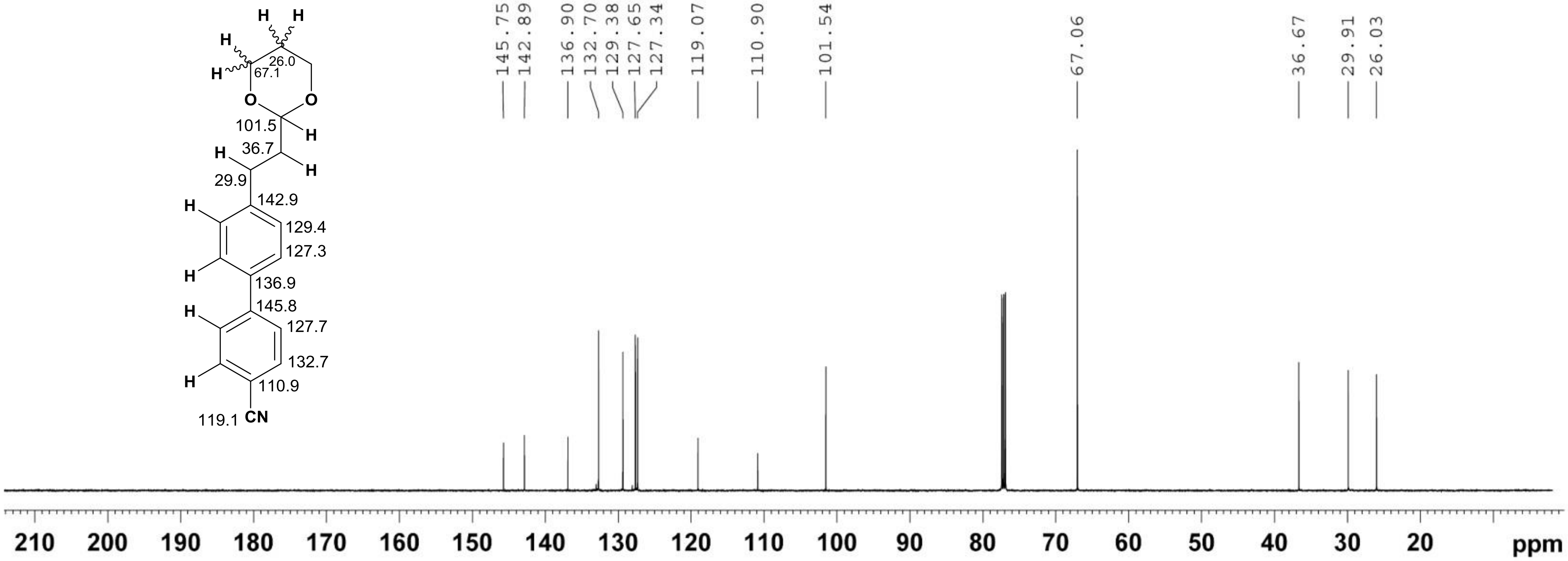

4'-Butyl-3'-methylbiphenyl-4-carbonitrile, ¹H (5ba)

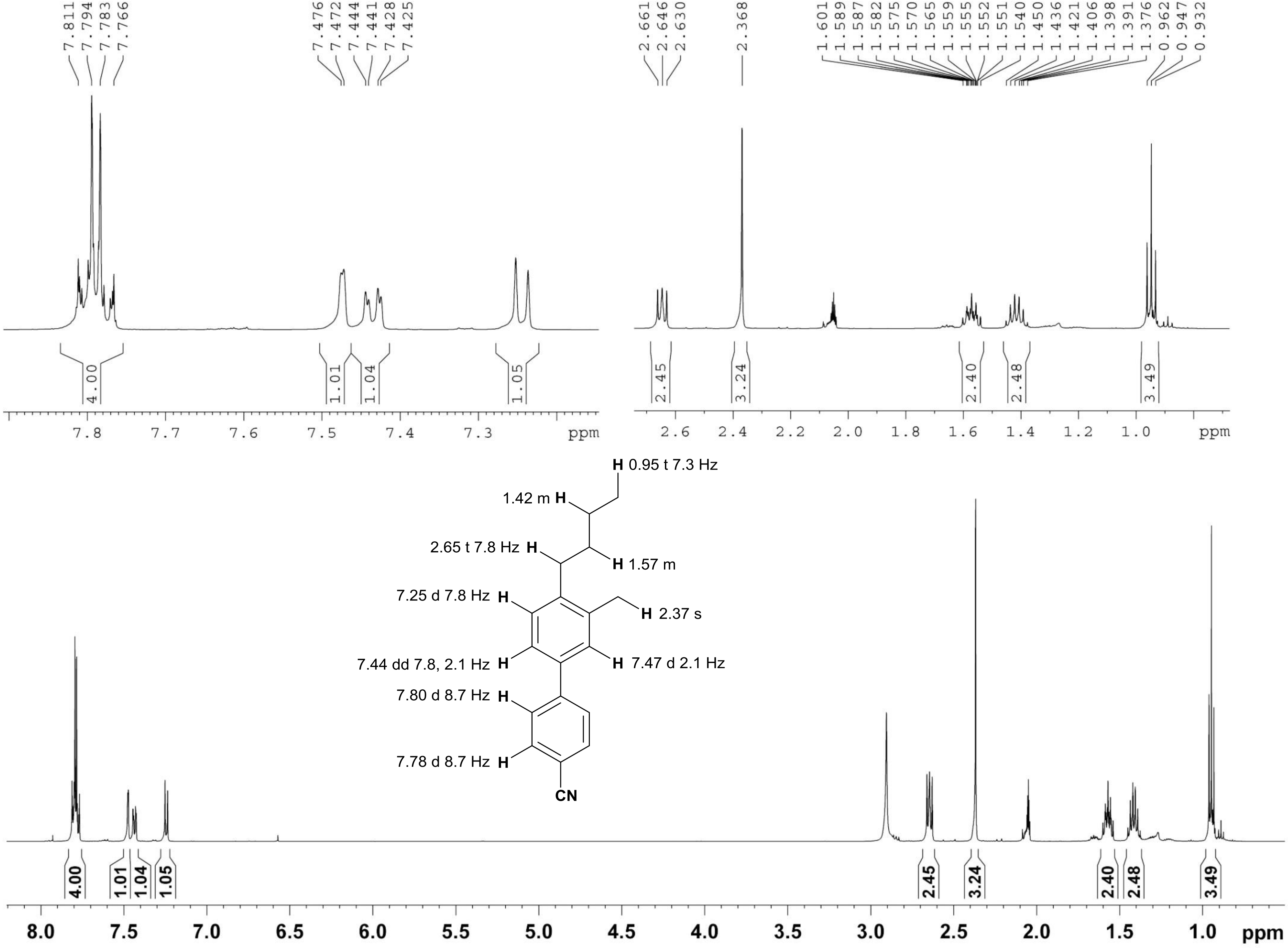

4'-Butyl-3'-methylbiphenyl-4-carbonitrile, ^{13}C (5ba)

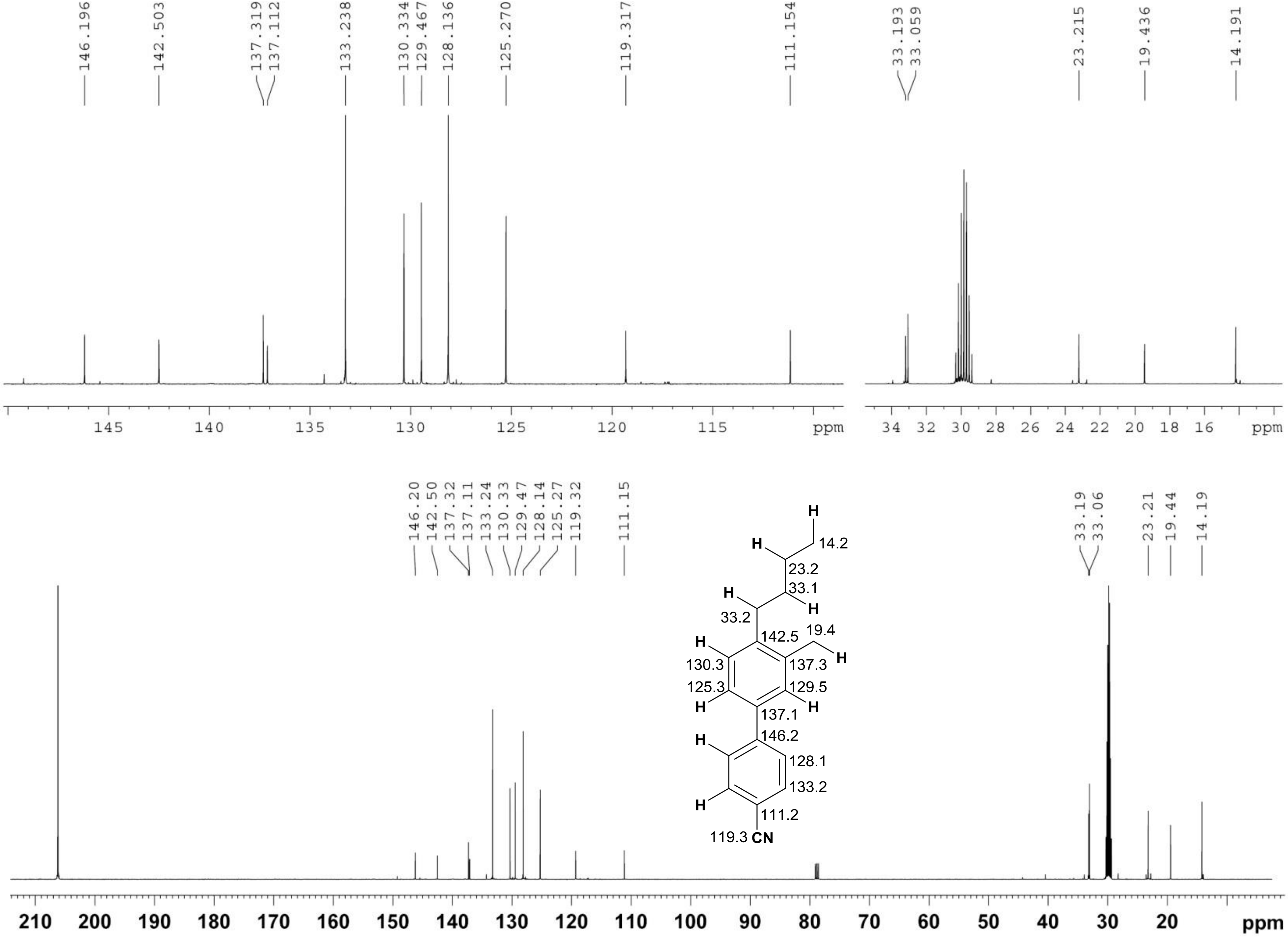

4'-Butyl-2'-methylbiphenyl-4-carbonitrile, ^{1}H (5ca)

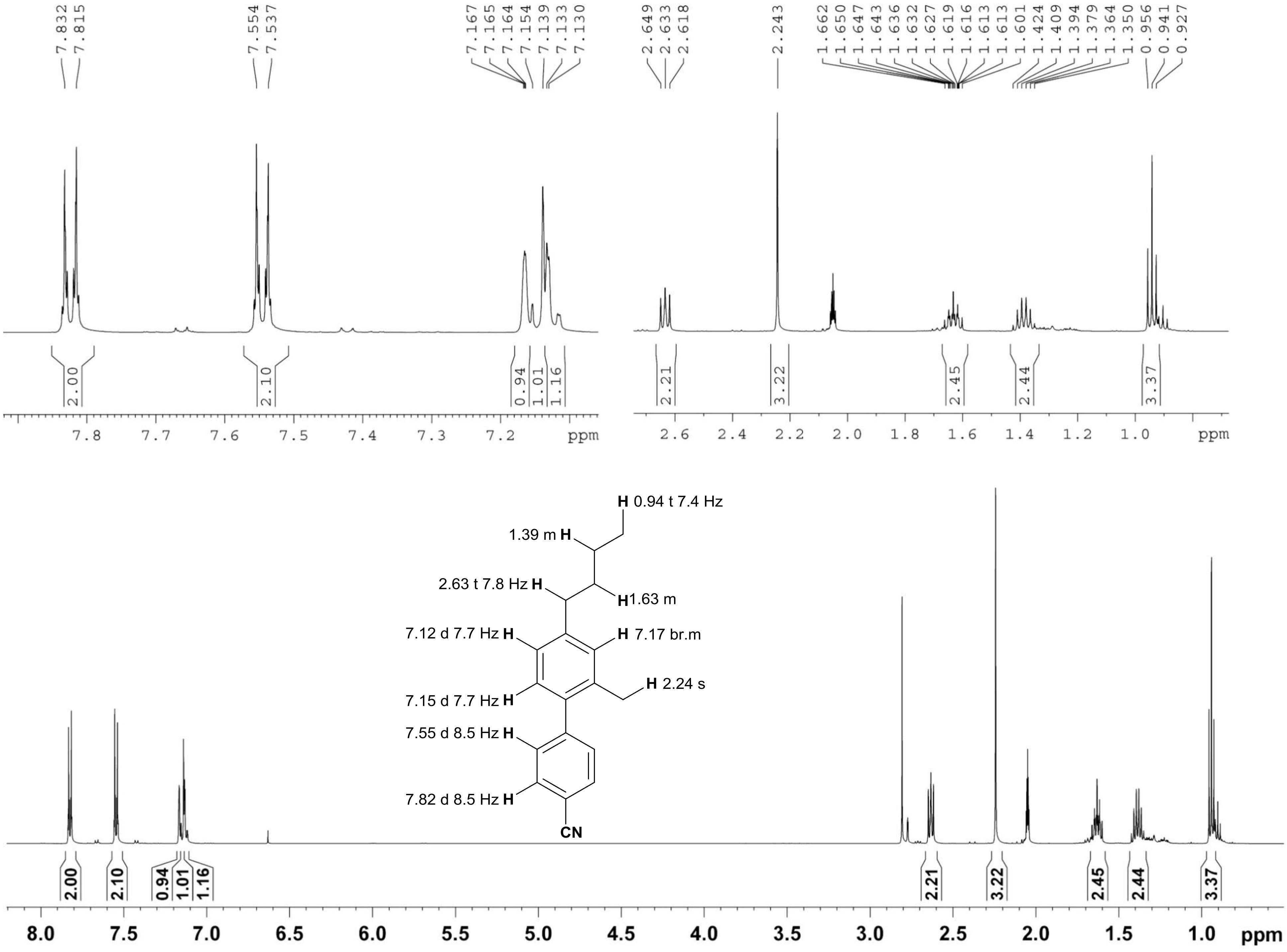

4'-Butyl-2'-methylbiphenyl-4-carbonitrile, ^{13}C (5ca)

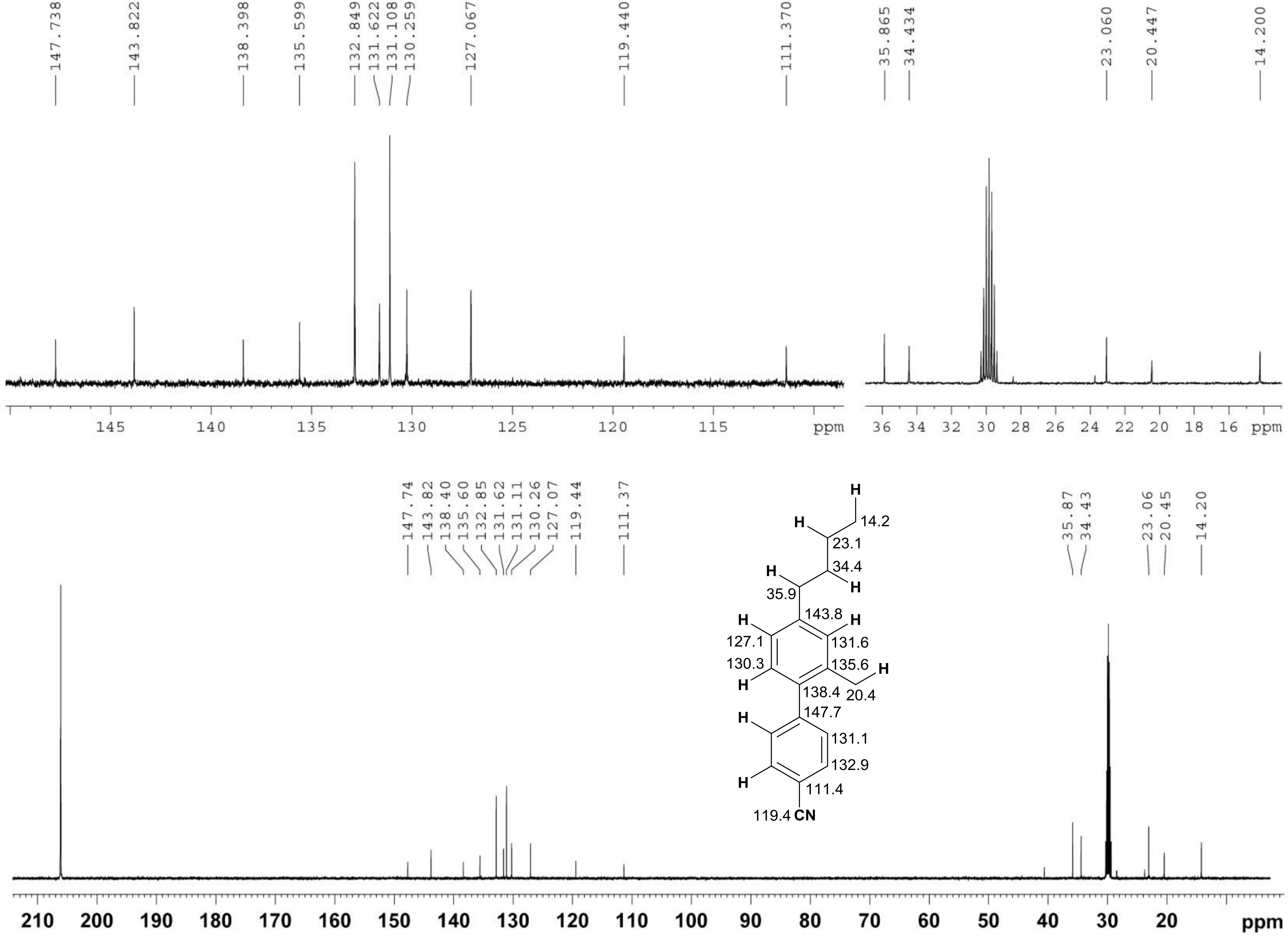

4'-Butyl-3'-methoxylbiphenyl-4-carbonitrile, ¹H (5da)

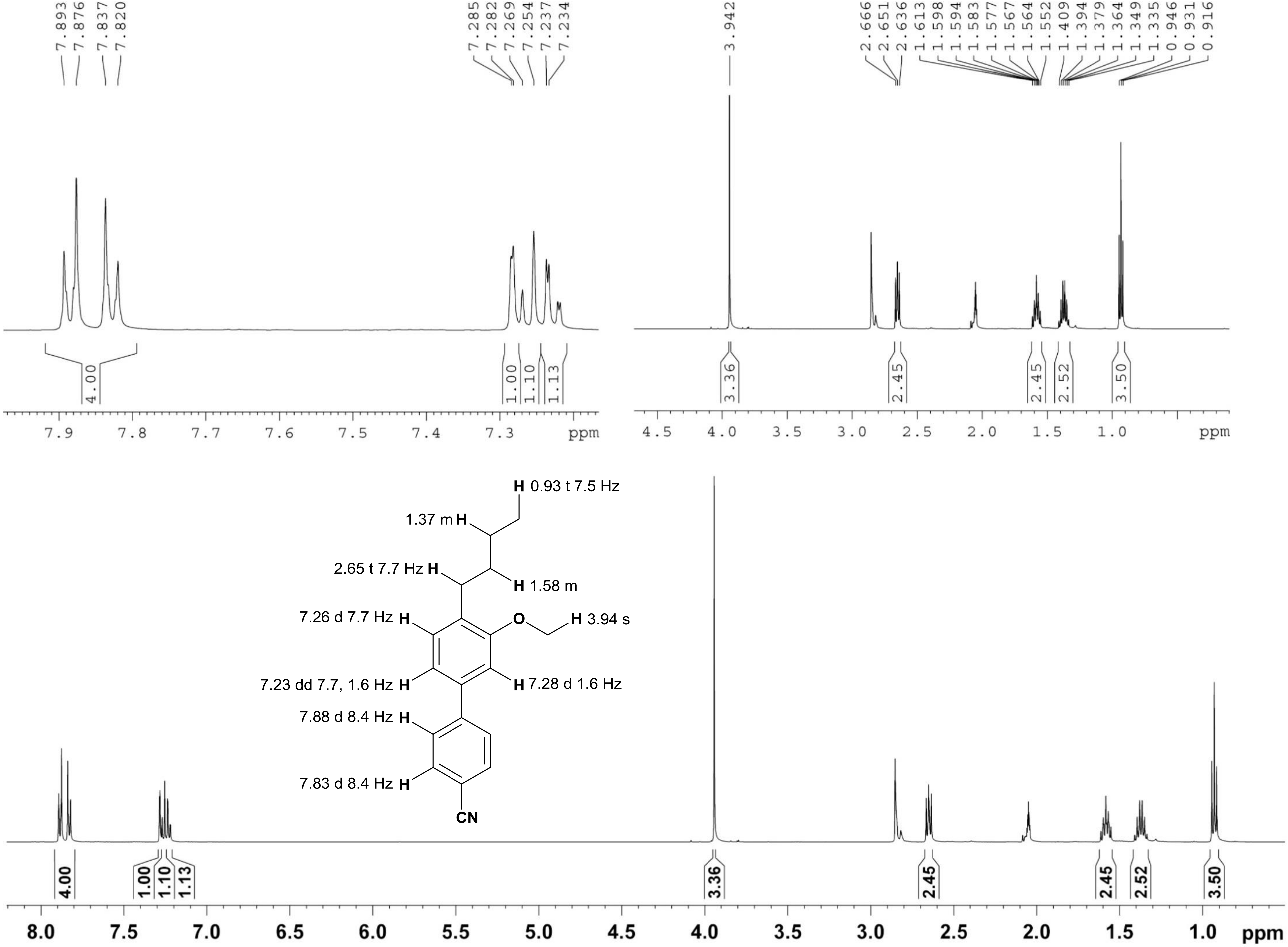

4'-Butyl-3'-methoxylbiphenyl-4-carbonitrile, ^{13}C (5da)

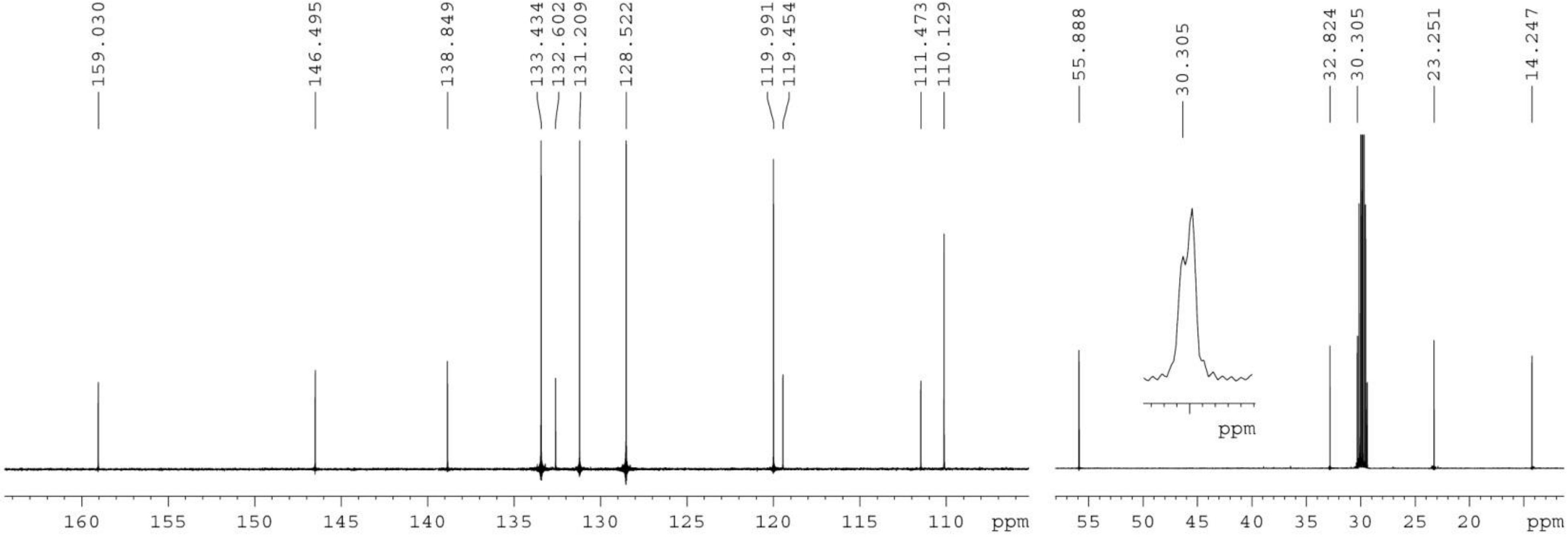

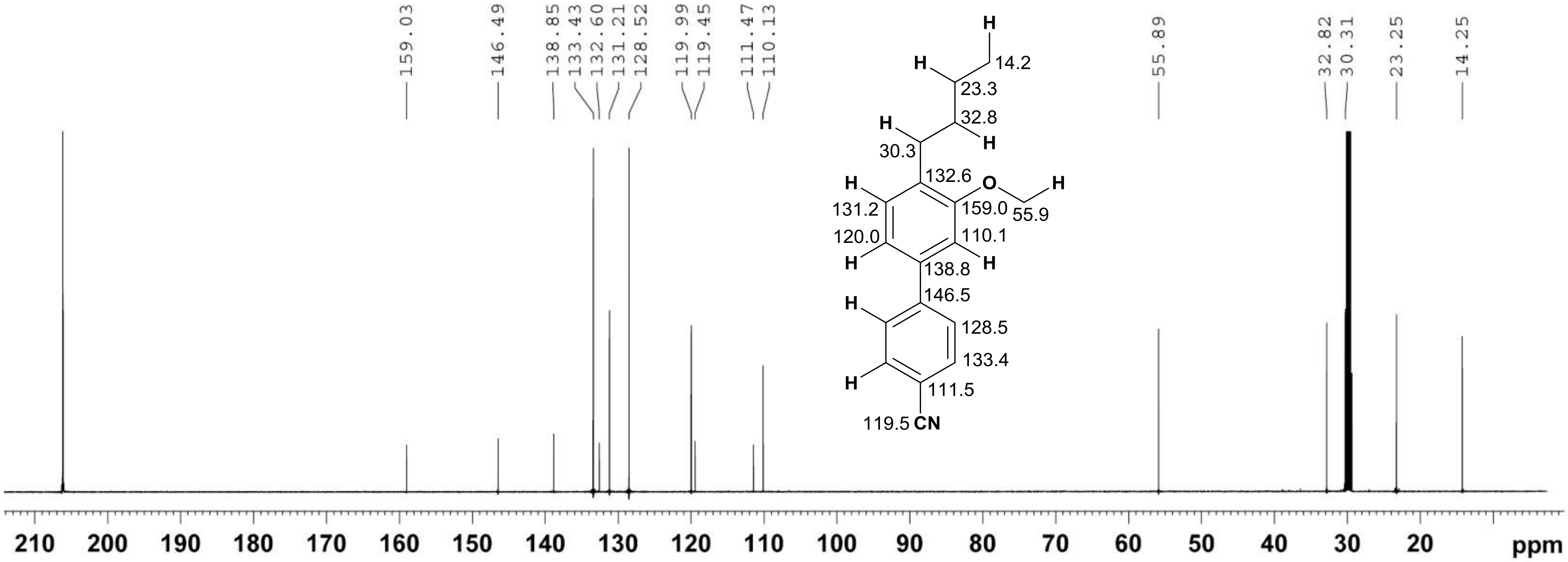

4'-Butyl-2'-methoxylbiphenyl-4-carbonitrile, ^{1}H (5ea)

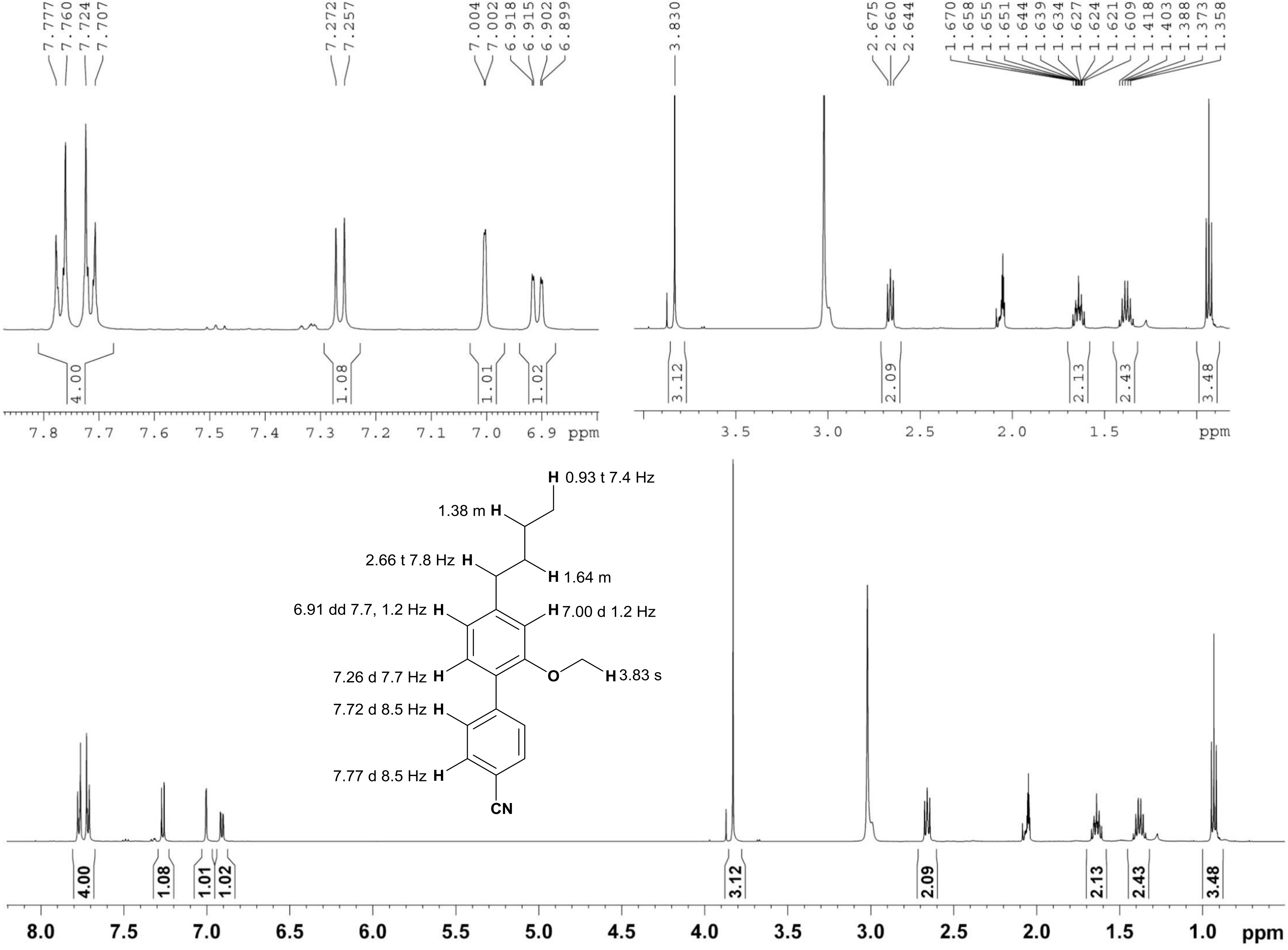

4'-Butyl-2'-methoxylbiphenyl-4-carbonitrile, ^{13}C (5ea)

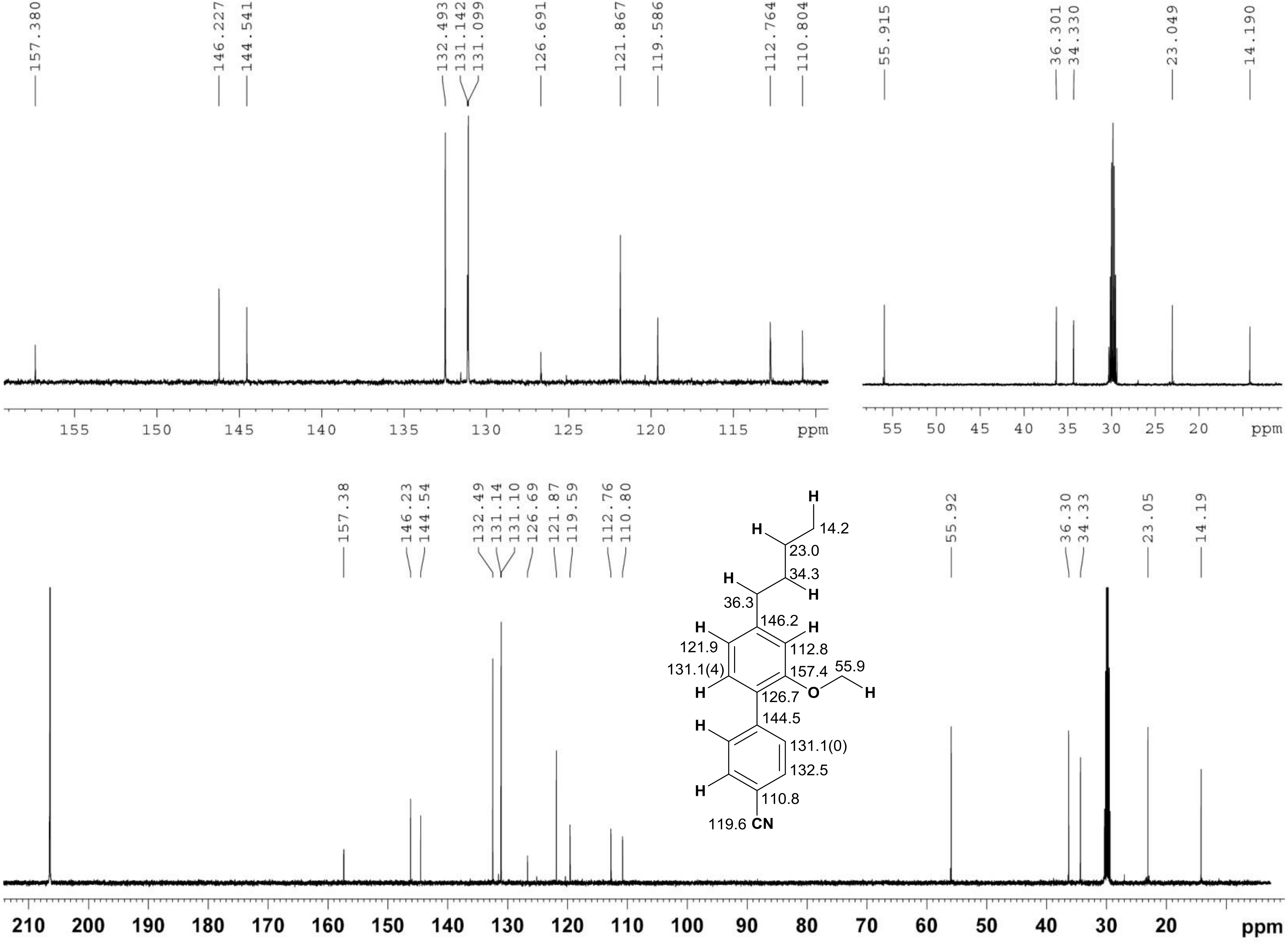

4'-Butyl-3'-fluorobiphenyl-4-carbonitrile, ^{1}H (5fa)

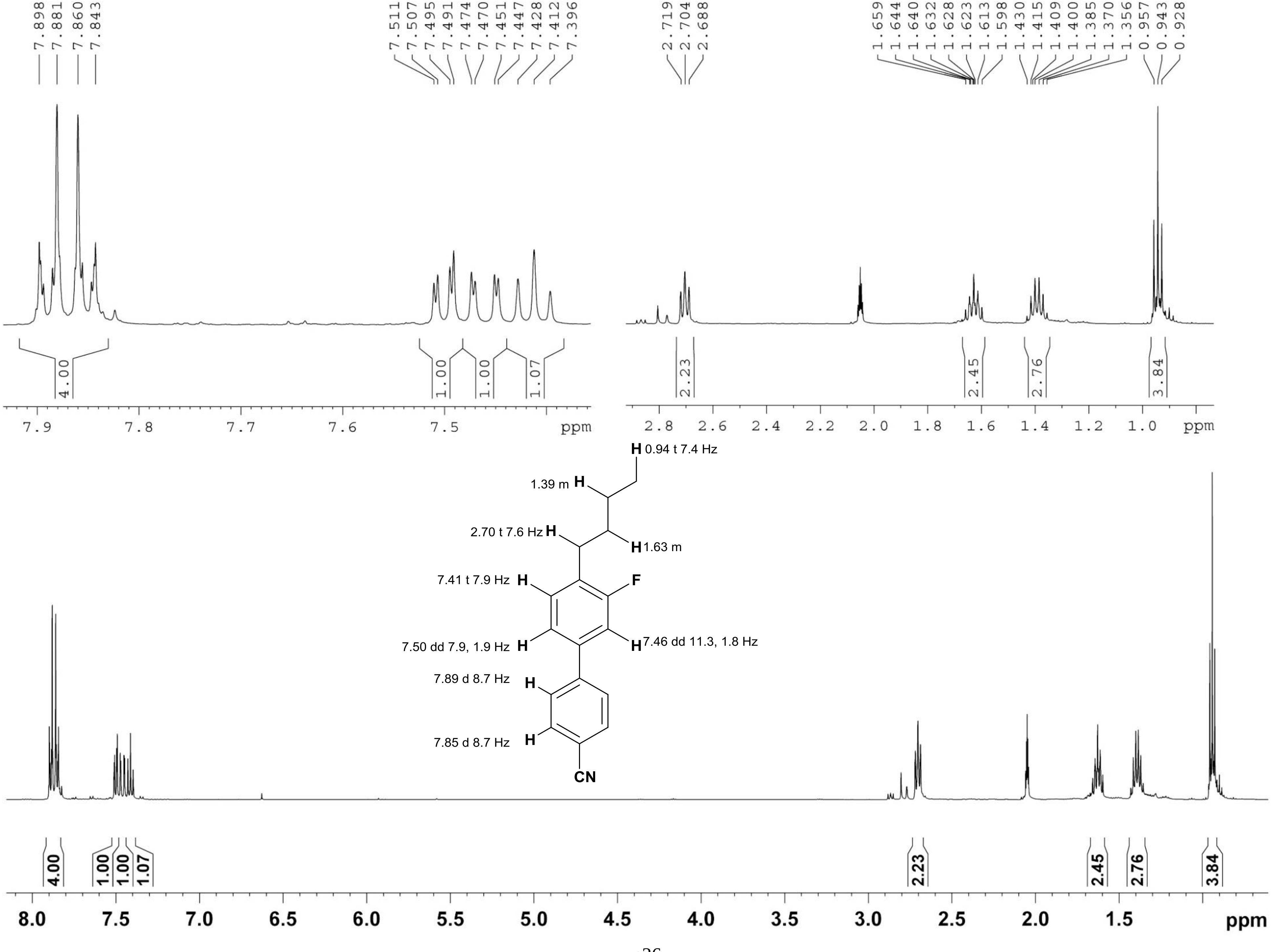

4'-Butyl-3'-fluorobiphenyl-4-carbonitrile, ^{13}C (5fa)

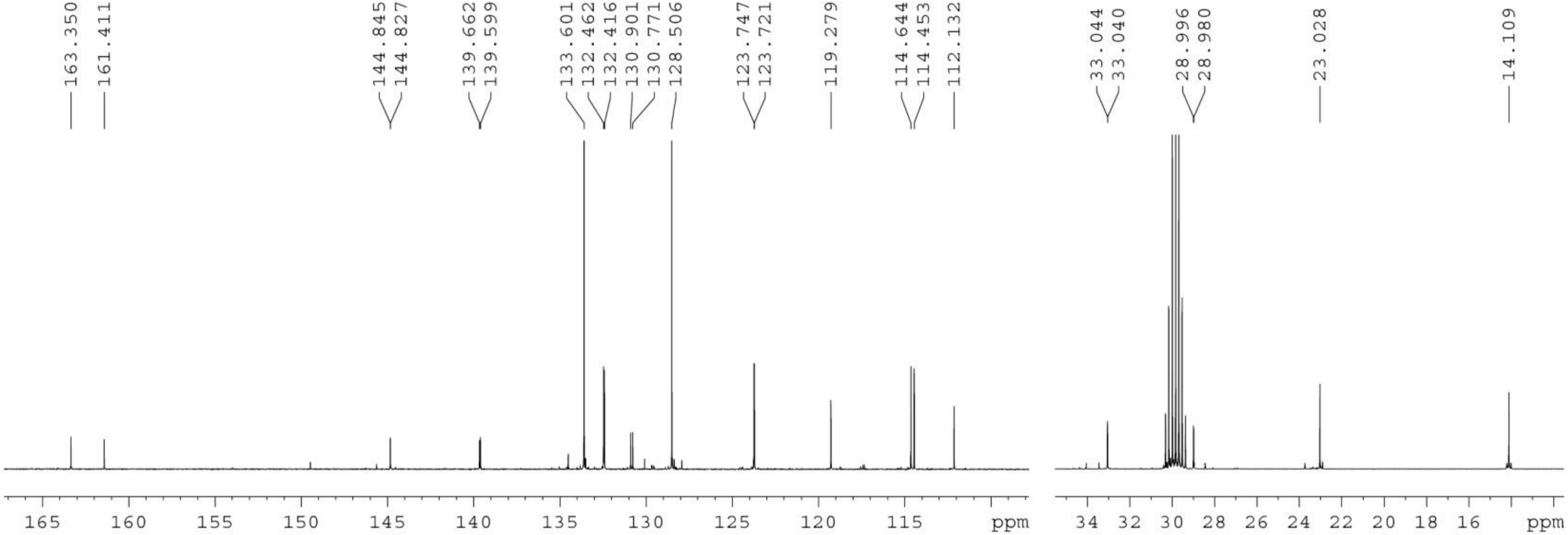

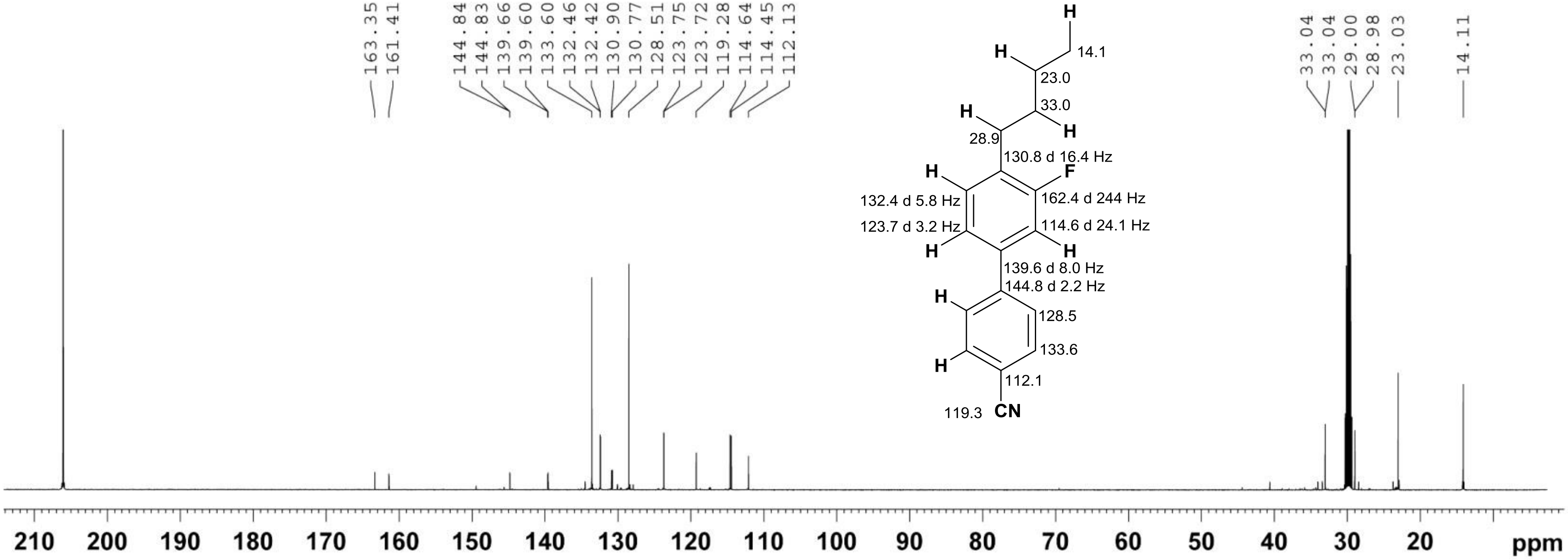

4'-Butyl-3'-fluorobiphenyl-4-carbonitrile, ^{19}F (5fa)

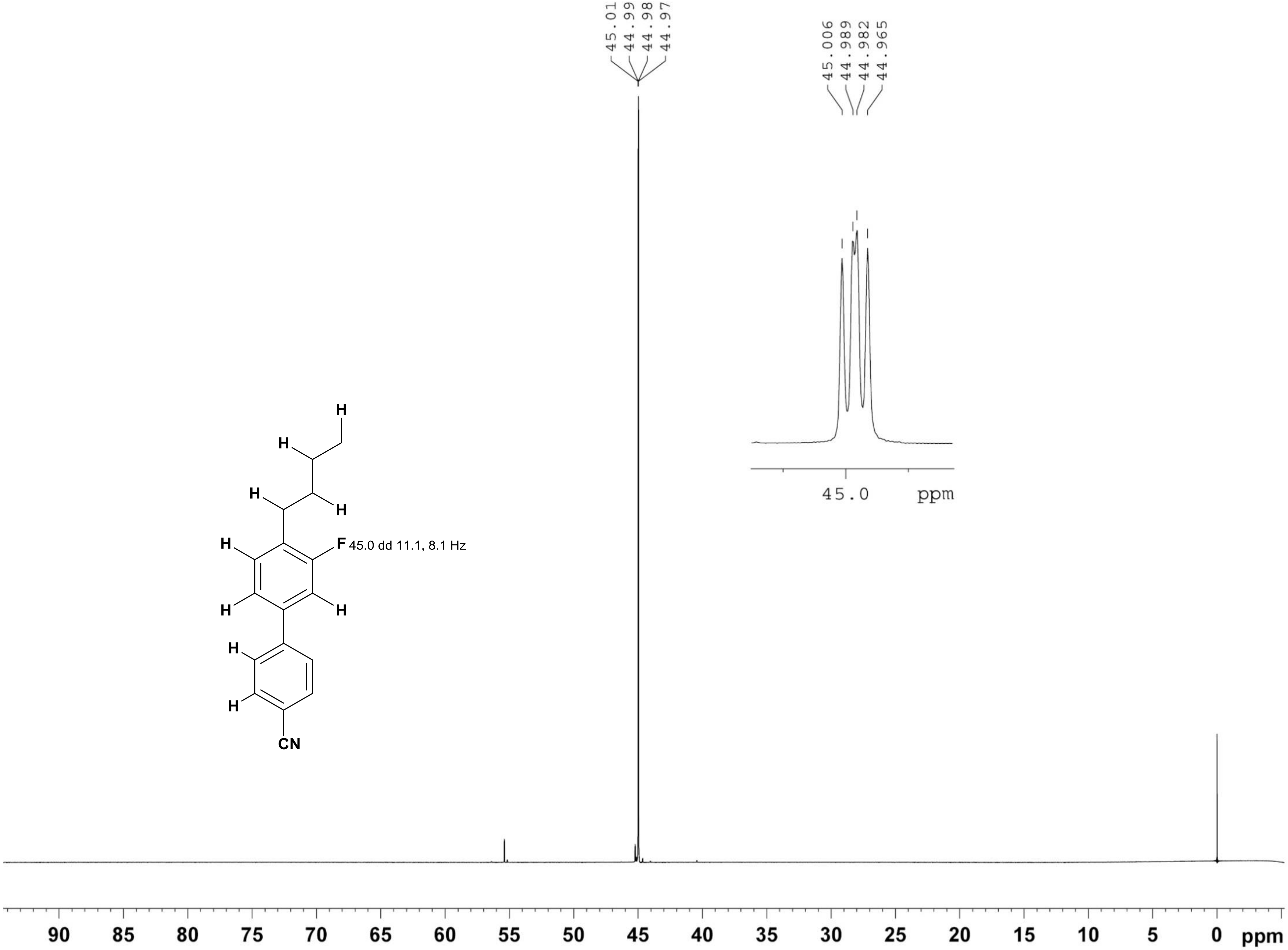

4'-Butyl-3',5'-difluorobiphenyl-4-carbonitrile, ^{1}H (5ga)

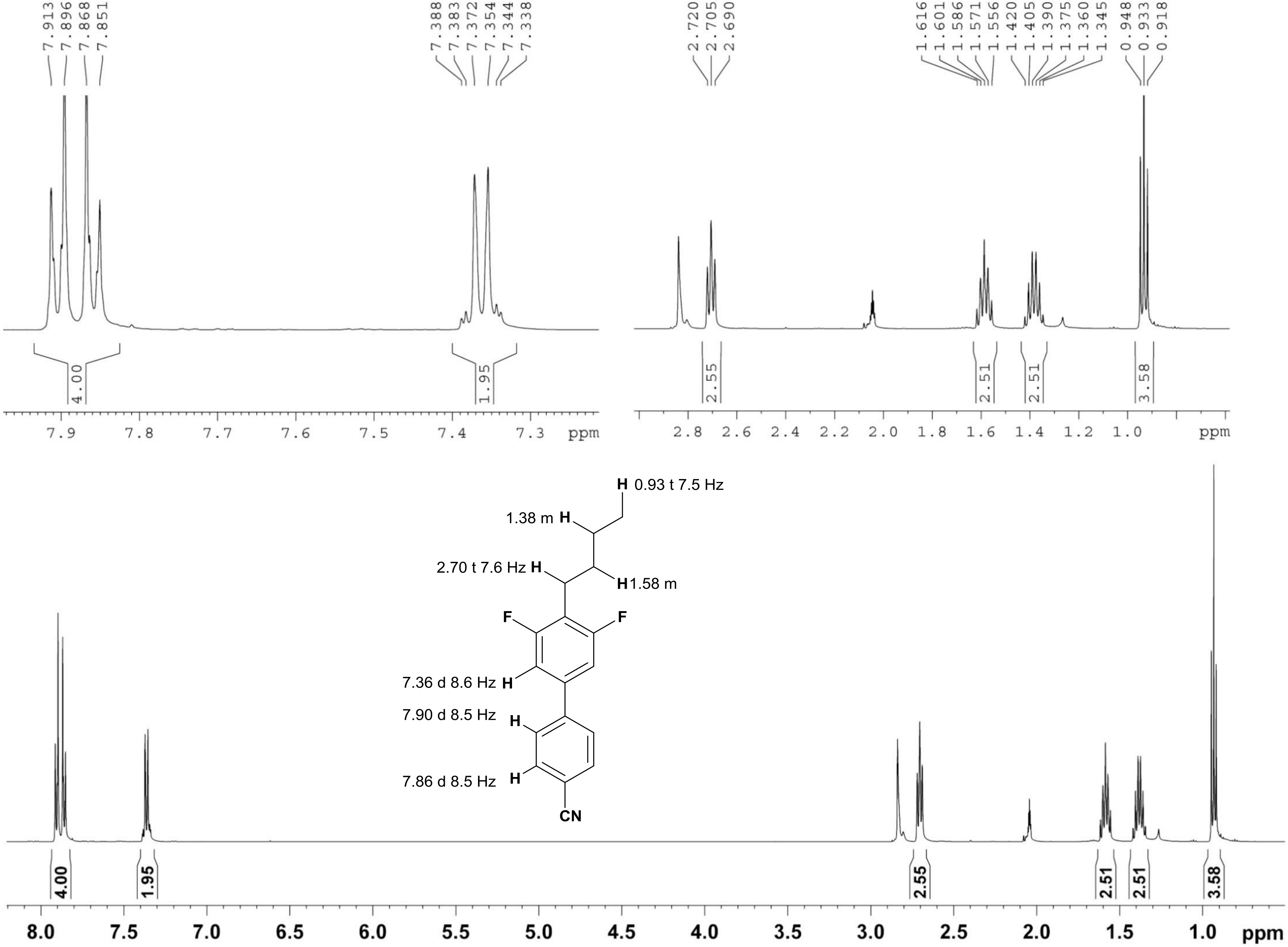

4'-Butyl-3',5'-difluorobiphenyl-4-carbonitrile, ^{13}C (5ga)

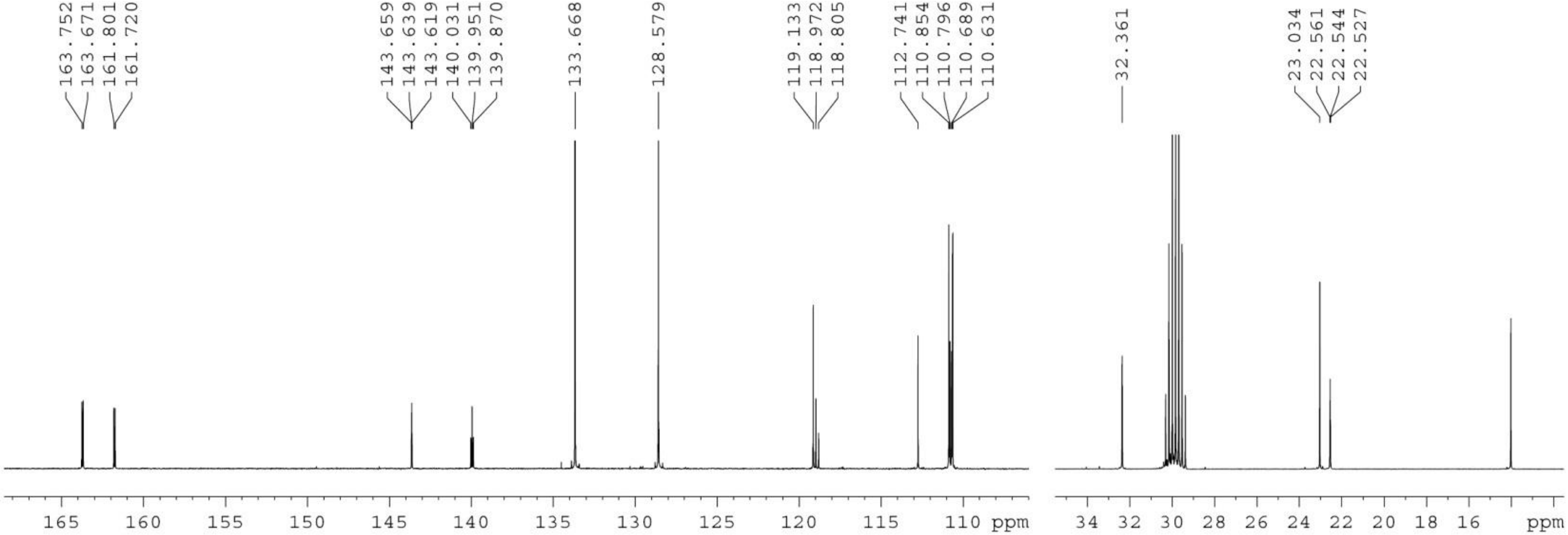

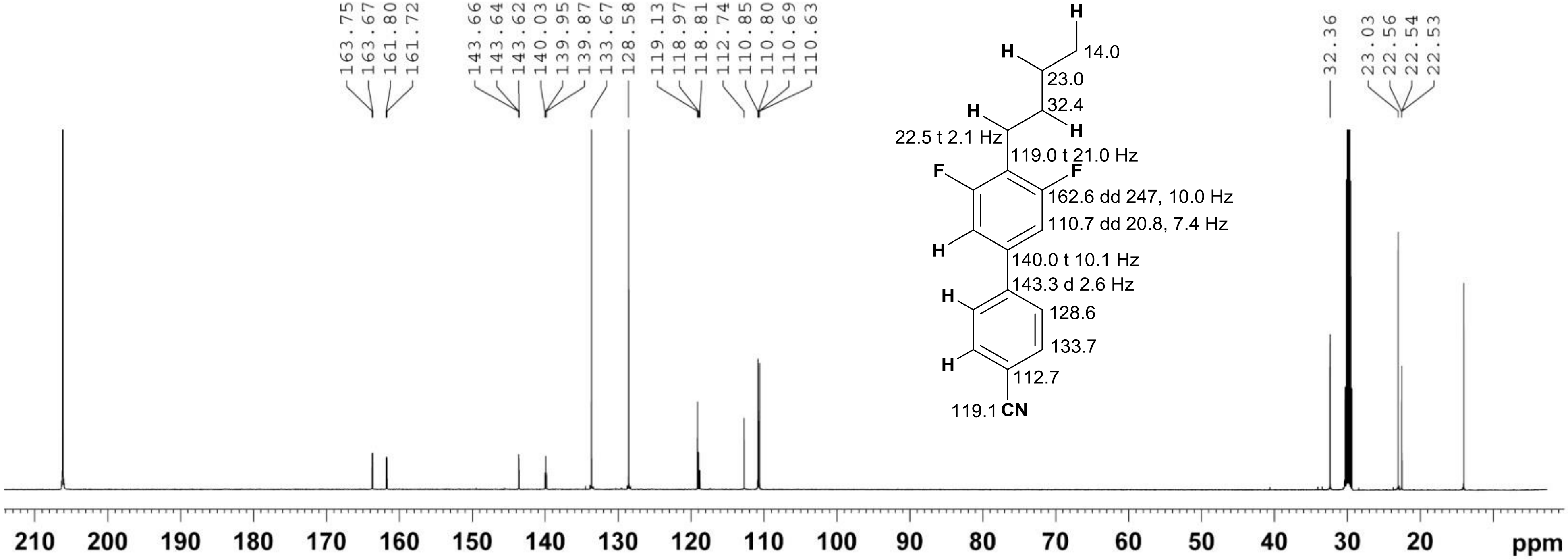

4'-Butyl-3',5'-difluorobiphenyl-4-carbonitrile, ^{19}F (5ga)

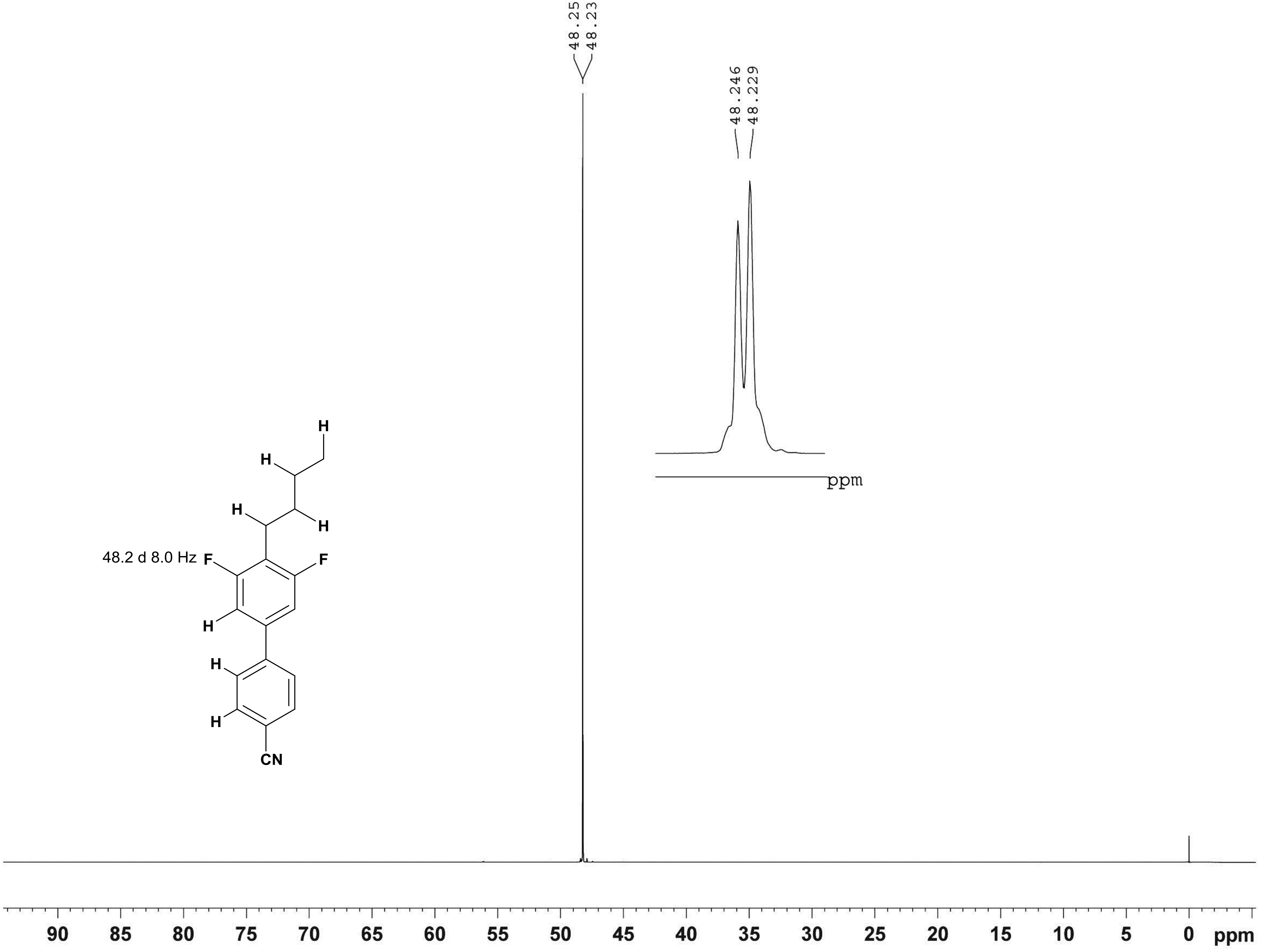

4'-Butyl-4''-methyl-[1,1':3',1''-terphenyl]-4-carbonitrile, ^{1}H (5ha)

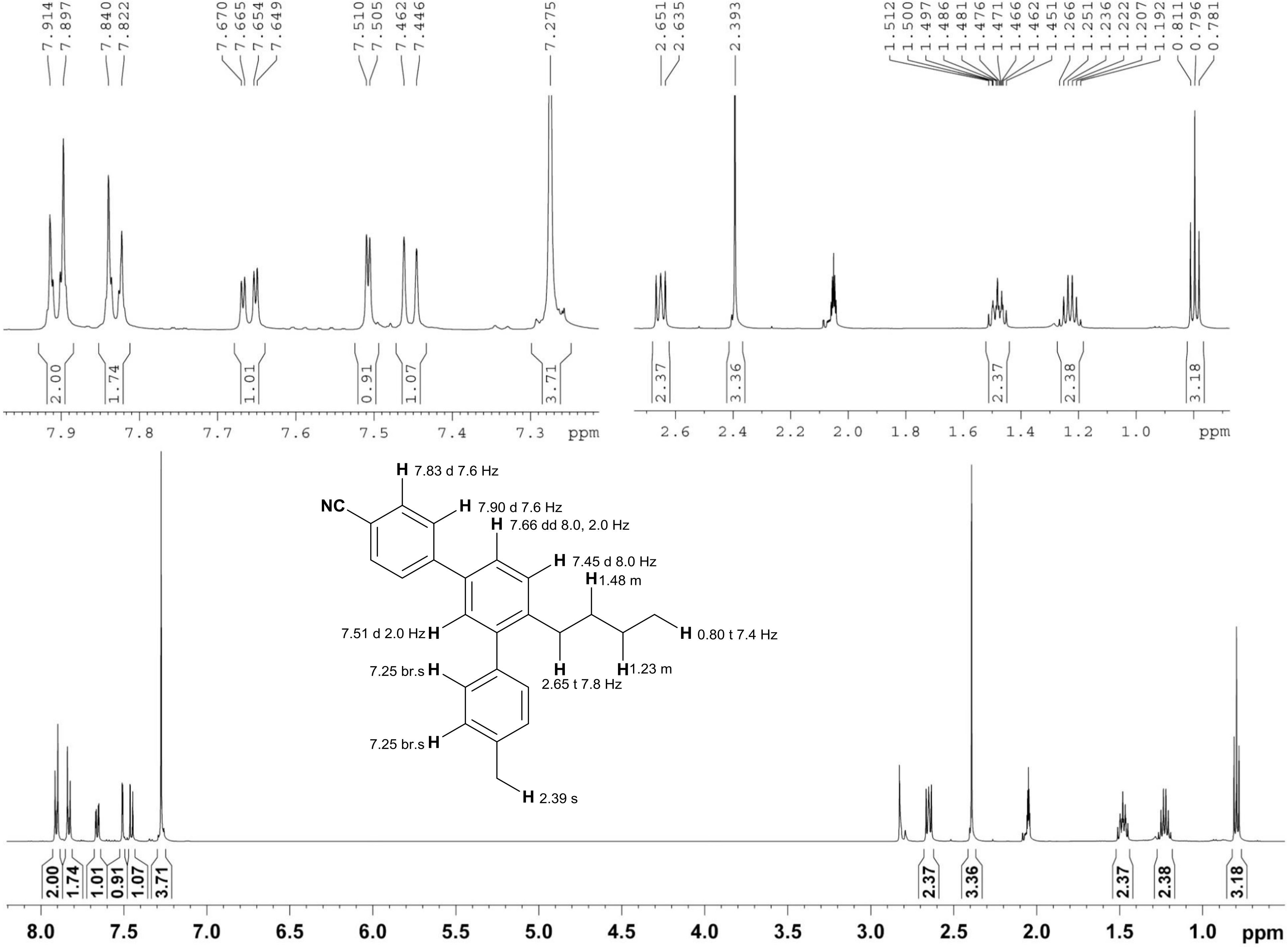

4'-Butyl-4''-methyl-[1,1':3',1''-terphenyl]-4-carbonitrile, ^{13}C (5ha)

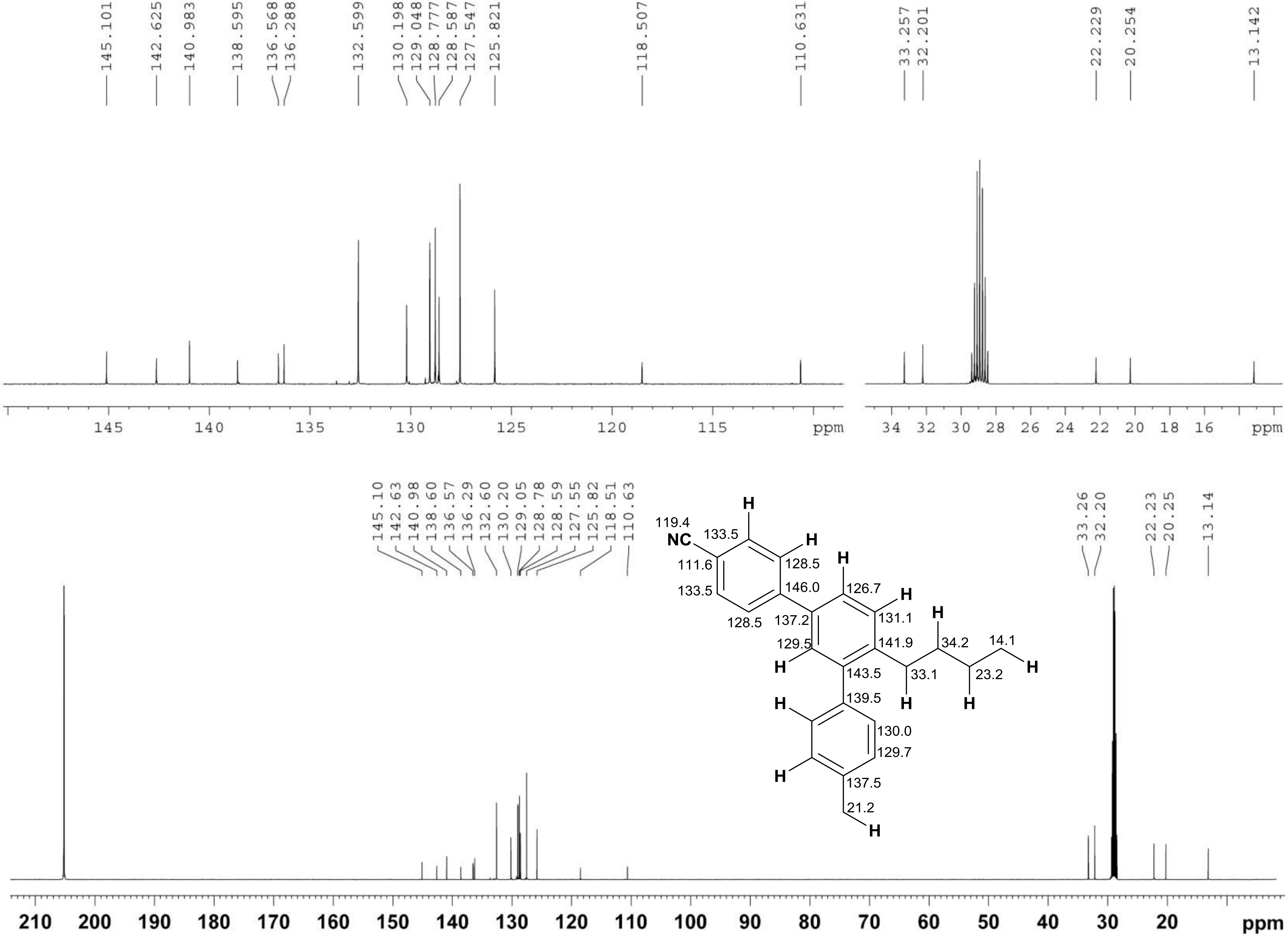

4-Butyl-1-(4-cyanophenyl)naphthalene, ^{1}H (5ia)

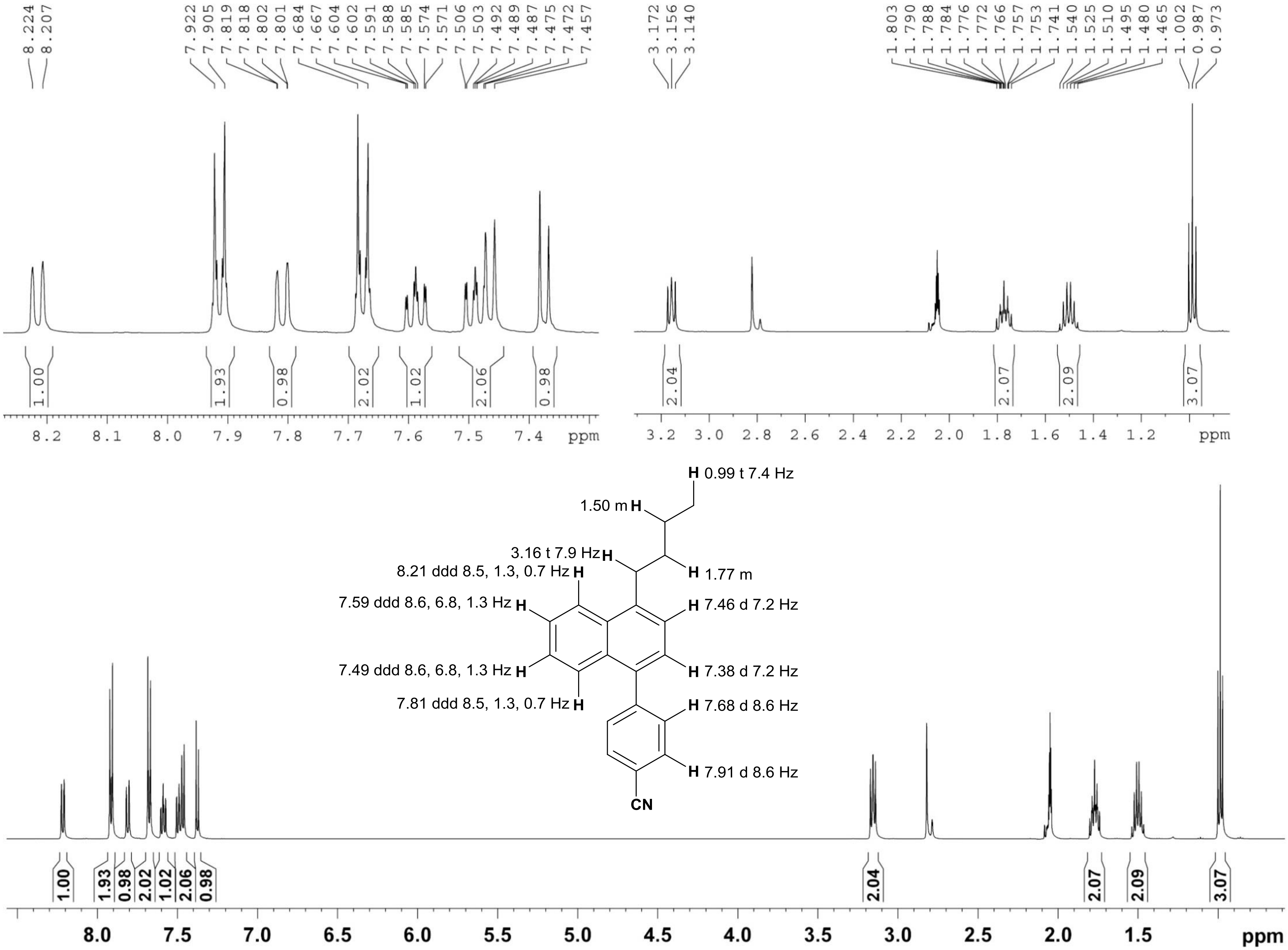

4-Butyl-1-(4-cyanophenyl)naphthalene, ^{13}C (5ia)

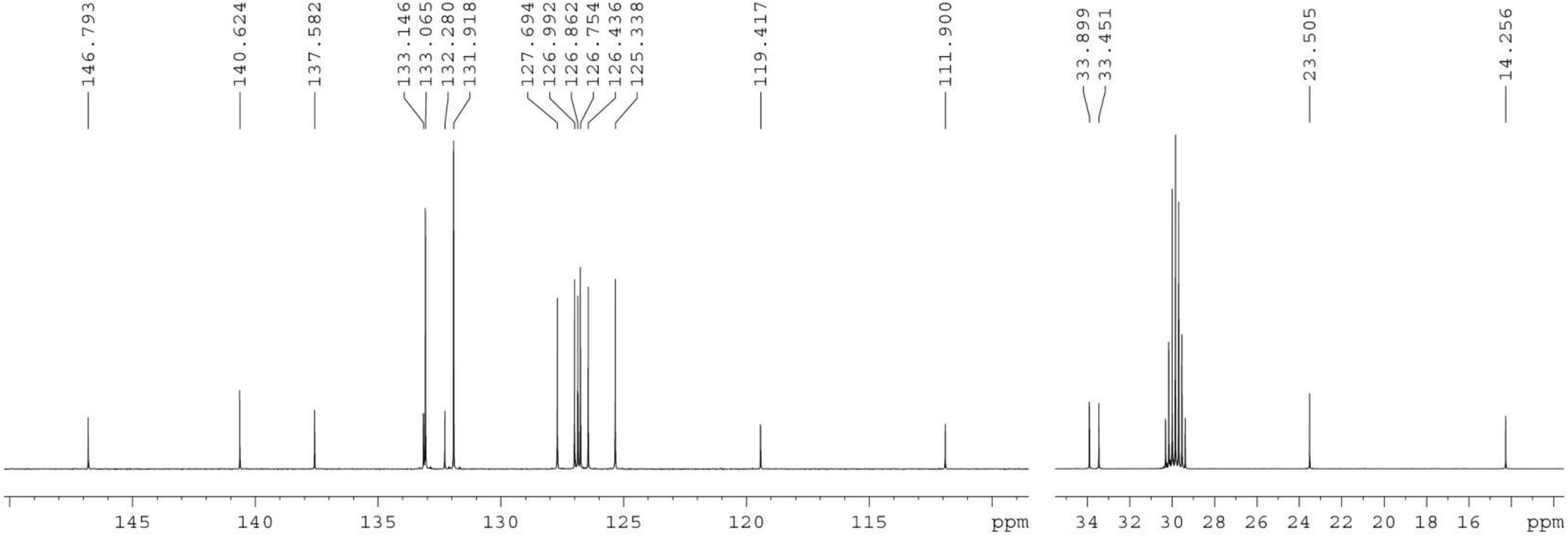

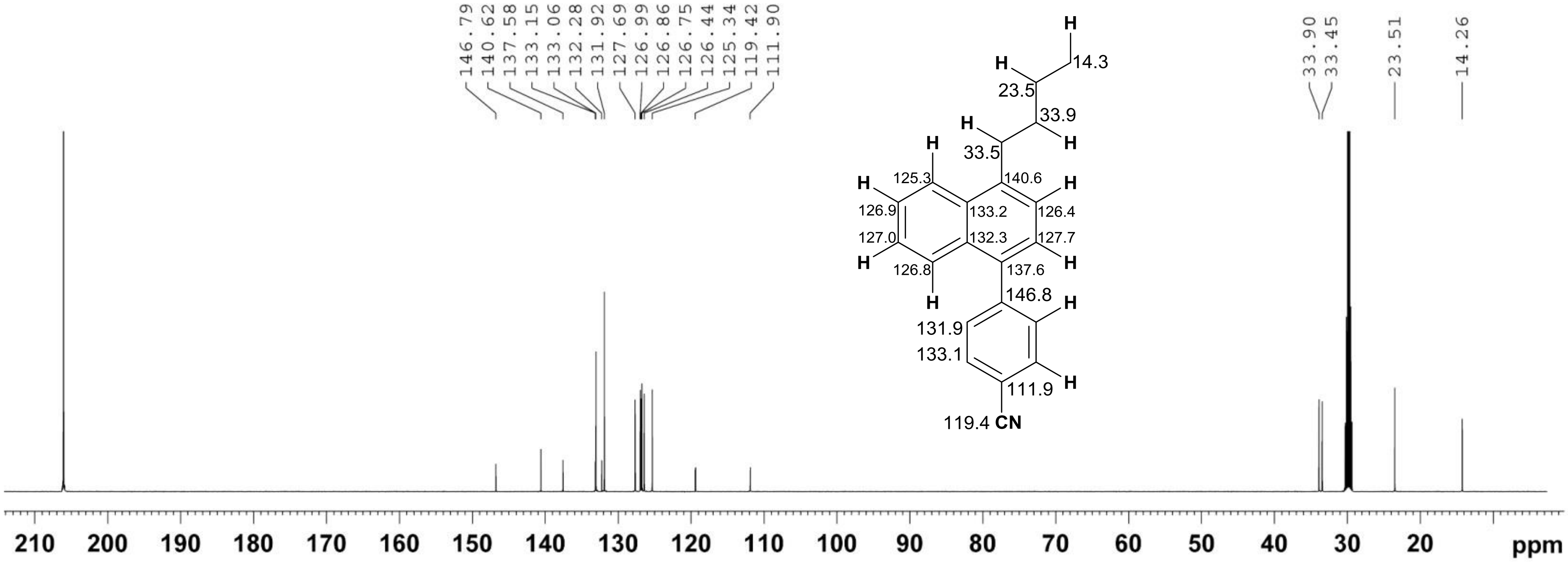

Supplement: File 1 — Experimental section and 1H, 13C and 19F NMR spectra of all synthesized compounds. [file Beilstein_J_Org_Chem-12-1577-s001.pdf]
